# Representative Genotyping, Recombination and Evolutionary Dynamics Analysis of *TSA56* Gene Segment of *Orientia tsutsugamushi*

**DOI:** 10.3389/fcimb.2020.00383

**Published:** 2020-08-05

**Authors:** Jiali Long, Yuehong Wei, Xia Tao, Peng He, Jianmin Xu, Xinwei Wu, Wei Zhu, Kuncai Chen, Zhicong Yang

**Affiliations:** Guangzhou Center for Disease Control and Prevention, Guangzhou, China

**Keywords:** *Orientia tsutsugamushi*, 56-kDa type-specific antigen gene, PCR method, genotyping, recombination, evolutionary dynamics

## Abstract

Scrub typhus is a zoonotic disease caused by *Orientia tsutsugamushi* (*O. tsutsugamushi*). *Orientia tsutsugamushi* has various genotypes and more new strains with difference in sequences increasingly appeared. Whether the accurateness of one special nested PCR method which amplifies segment instead of entire open reading frame (ORF) sequence meets the current work of identifying new strains and classifying genotypes remains to be confirmed. And the origins and evolution of this organism have not been thoroughly elucidated. Accordingly, in this study, segments and the entire ORF of the 56-kDa type-specific antigen (*TSA56*) gene of *O. tsutsugamushi* were collected, including 209 clinically isolated strains in Guangzhou, China from 2012 to 2016 and 139 reference strains worldwide. By performing phylogenetic analysis, we proved that the accurateness of the particular PCR method which almost met detection need. This re-grouping result showed that segments perfectly represented and identified strains of Karp, Boryong, Gilliam, TA763, Kawasaki and part of Kato genotype, and this accuracy is not restricted by region and time. Sequence diversification of Shimokoshi and some Kato strains made their genotyping need to consider entire ORF sequences, but their weak recognition might not be due to recombination. The frequent genetic recombination and high point mutations contributed to genetic diversification of the *TSA56* gene. Major overlapping regions of most recombination events occurred between strains of the same genotype, especially Karp and Kato genotype. And cross-genotype overlapping events occurred between Karp and Boryong/Gilliam/TA763/Kato, Kato and Kawasaki/Gilliam/TA686, Boryong and TA686, and Gilliam and Kawasaki. But Segment has quite low recombination frequency and stable mutation trend from 1943 to 2016. So segment is a relatively conserved part of the *TSA56* ORF as for its stable trend of genetic diversity, and it may anchor and represent the entire *TSA56* ORF gene. And genetic diversity is rejected as one potential reason for the increased incidence of scrub typhus. But an occasional recombination event created an unrecognized genotype which might be due to the breakage of VD II and AD II. Additionally, strains in Guangzhou were homologous and Karp genotype was detected as a dominant.

## Introduction

Scrub typhus is a zoonotic disease caused by *Orientia tsutsugamushi* (*O. tsutsugamushi*) infection, resulting in major public health problems worldwide. Scrub typhus is considered to be an acute infectious disease with an incubation period of 6–21 days, and generally its clinical manifestations are a wide range of non-specific clinical symptoms including headache, fever, rash, breathlessness, cough, nausea, vomiting, myalgia, local lymphadenopathy, and distinctive eschars at the inoculation site (Diaz et al., 2018). Seven to fifteen percentage of cases may develop fatal multiple-organ involvement within 2 weeks after infection, like acute respiratory distress, diffuse endothelial infection, diffuse interstitial pneumonia, liver lesions, acute renal failure, myocarditis and encephalitis (Mendell et al., 2017; Diaz et al., 2018; Soong, 2018). Previous records have revealed that scrub typhus is endemic in vast regions from Russian Far East in the north to northern Australia in the south and Afghanistan in the west, including insular territories of the Pacific and Indian Oceans (Kelly et al., 2009; Paris et al., 2013). In some endemic areas, scrub typhus is the main cause of non-malarial fever (Soong, 2018). More than 20% of hospitalized patients with acute undifferentiated fever are diagnosed with scrub typhus infection in rural areas of endemicity (Suttinont et al., 2006). More than one million cases of scrub typhus are estimated to occur worldwide each year, and approximately one billion people are at risk (Watt and Parola, 2003). The mortality rate is estimated 10% unless treated, very likely resulting in more deaths than dengue, while appropriate treatment can reduce rate to ~1–5% with poorer outcomes observed in the elderly, pregnant women and those with complications (Paris et al., 2013; Zhang, 2013). However, the global burden of this infectious disease may be underestimated. At present, while its endemic areas continue to broaden in Asian regions, strong molecular, serological and clinical evidence suggested that this disease has spread in Africa and South America (Diaz et al., 2018). And the incidence of this disease is increasing worldwide (Yang and Zhao, 2008; Tilak and Kunte, 2019). Currently, cases of vertical transmission through the mother to the baby have been observed (Suntharasaj et al., 1997). The World Health Organization has declared scrub typhus one of the most underdiagnosed/underreported diseases in the world, and stressed the necessity to better understand its vectors, outbreaks and pathogenesis associated with this potentially fatal organism both within and beyond its endemic areas (Luce-Fedrow et al., 2018).

*O. tsutsugamushi* is a Gram-negative, obligate intracellular bacteria known as the etiological agent of scrub typhus with many strains. The genotyping method based on the 56-kDa type-specific antigen (*TSA56*) gene has been widely recognized as an effective approach for genotyping *O. tsutsugamushi* (Kelly et al., 2019). In recent decades, new genotypes have been continuously discovered and confirmed, and more researches have been conducted on the biological characteristics of *TSA56*. *TSA56* protein is a major type-specific antigen (TSA) of *O. tsutsugamushi*. *TSA56* antigen gene has an open reading frame (ORF) of ~1,600 nt in length and encodes ~530 amino acids (aa). The length of the coding region and the number of encoded amino acids is slightly different between strains. This protein is mainly different in the four variable domains (VD) with spans of 16–40 aa in the hydrophilic regions, I through IV. VDs are responsible for the considerable degree of antigenic variation and define genotypes of *TSA*56 (Ohashi et al., 1992). The nested PCR detection targeting *TSA*56 gene is highly specific and generally entire ORF sequence of TSA56 gene is the target. But the method for entire ORF sequence is costly and requires high training resources. Besides, TSA56 is full of point mutations and recombination, which may affect detection sensitivity because the sequence variability of the gene may affect primer annealing (Paris et al., 2013). Therefore, that high resource costs and affected sensitivity limit the popularity of this method. Yang et al. (2012) used two pairs of primers for the TSA56 gene and amplified a segment of 418–453 nt in length which covers the antigenic domain II (AD II) and VD II for genotyping, instead of amplifying the whole length. This segment located at around No. 405–894 nt (No. 135–298 aa) in the whole 1,600 nt, which simplified the PCR process and reduced the cost. Guangzhou center for disease control and prevention applies this method to monitor cases, hosts and chigger mites through Scrub Typhus and Rodent-borne Disease Surveillance System for several years. Since the new strains with difference in sequences increasingly appeared, whether the accurateness of this method satisfies the work need to be proved.

More than 40 serotypes or antigenic strains of *O. tsutsugamushi* been isolated worldwide (Tilak and Kunte, 2019). The most common genotype is Karp, following by Gilliam (Kelly et al., 2009; Tilak and Kunte, 2019). Approximately 50 and 25% of human infections are related to Karp and Gilliam strain infections, respectively (Soong, 2018). The TSA56 gene sequence of different strains has homology to some extent, and differences (Ohashi et al., 1992). Sonthayanon et al. (2010) proved that 25% of patients were infected with multiple strains of *O. tsutsugamushi*. Some scholars suggested that the multiple antigenic variants of *O. tsutsugamushi* might be evolved from genetic recombination among different strains and point mutations, when the strains co-parasitized in chigger mites. Although *O. tsutsugamushi* has many genotypes, and more genotypes have been continuously published, few research has evaluated the genetic diversification and dynamic characteristics of *TSA56* between genotypes in China to better understand this gene such as its structure and function mechanism. Furthermore, although some studies on strains in other countries/regions compared the full-length sequences of *O. tsutsugamushi* using phylogenetic analysis, the authenticity of these phylogenetic analysis on sequences with frequent recombination needs verification (Wongprompitak et al., 2015). And the differences between various strains from different years and regions have yet to be studied.

Accordingly, in this study, we collected strains from Guangzhou in 2012–2016 through surveillance system and performed analysis with reference strains worldwide. we planned to process all reference strains with at least 80% ORF sequences (in the following analysis we simplified to call these strains as “the strains with entire ORF sequences”) into segments located near 405–894 nt and to re-group all segments of Guangzhou sample strains and reference strains by phylogenetic analysis. We compared the re-grouping results with the genotypes of strains based on entire ORF sequences to test whether this PCR method was accurate enough to meet the need for disease detection and whether this segment was representative of the *TSA56* gene. We further assessed the basic composition, genetic diversification analysis and evolutionary dynamics analysis of the strain segments to understand *TSA56* gene. Among them, we analyzed the grouping, homology and differentiation, and the dominant strains of Guangzhou strains.

## Materials and Methods

### Sample Collection and DNA Extraction

From September 2012 to December 2016 in Guangzhou, whole blood specimens of scrub typhus patients diagnosed at sentinel hospitals and wild rat spleen specimens were collected. Specimens was later pretreated referring to Chen et al. (1998), and kept at −30°C for future use. The QIAamp Blood DNA MiniKit (QIAGEN) kit was used to extract total DNA of 200 μl blood samples and 20 μl pretreated rat spleen specimens, and DNA was stored at −70°C until use.

### PCR Amplification

Two pairs of primers for the complete ORF sequence of the *TSA56* protein gene and two pairs for the segment were designed and synthesized by Shanghai Yingweijieji Biotechnology Company, as shown in Supplementary Table 1 (Yang et al., 2012). PCR amplification was conducted in a 50-μL volume using Go TaqG2 Hot start colorless master mix (TaKaRa) kit.

The segment sequence of the *TSA56* protein gene: The first round of reaction was performed with tsu34 and tsu55 primers with 2 μl DNA template. Specific segments were amplified using 2 min at 94°C, 30 cycles: 30 s at 94°C, 1 min at 55°C, 2 min at 72°C, and finally at 72°C for 10 min. Then we took 2 μl of the product from the first round as the template to perform the second round of PCR with tsu10 and tsu11 specific primers. The steps were same as the first round. The PCR product of the second round was analyzed by QIAxcel Advanced automatic real-time capillary electrophoresis system, and the 500 bp positive sample was selected and sent to Shanghai Invitrogen Biotechnology Company for sequencing.

The complete ORF sequence of the *TSA56* protein gene: The first round of reaction used primer ST-A and primer ST-B to amplify the first half of the *TSA56* gene. Reaction conditions are 2 min at 94°C, 30 cycles: 1 min at 94°C, 1.5 min at 55°C, 2 min at 72°C, and finally 7 min at 72°C. The second round of reaction used primer ST-C and primer ST-D to amplify the second half of the 56-kDa gene. The amplification method was similar with the first round, instead the annealing time in the loop was shortened from 1.5 to 1 min. Positive samples of two rounds were selected and sent to Shanghai Invitrogen Biotechnology Company for sequencing.

Later, the sequenced nucleotide sequence was spliced with Bioedit software and analyzed with MEGA X software.

### Phylogenetic Analysis

To re-group 348 strains and then to test the accurateness of PCR method, we perform phylogenetic analysis. An optimal nucleotide substitution model and relative parameters were firstly required for this analysis using IQTREE software. IQTREE is an efficient, rapid, versatile program with multiple models for optimal nucleotide substitution model selection and is excellent for analyzing large datasets (Trifinopoulos et al., 2016). Smaller BIC values were associated with more appropriate substitution models. Then, a phylogenetic tree was established in phylogenetic analysis with 348 segment sequences with Mrbayes software to analyze similarities among sequences, and to re-annotate groups (Ronquist and Huelsenbeck, 2003).

### Recombination Analysis

RDP4 software (Martin et al., 2010) was implemented to detect potential recombinant sequences and parental sequences in the gene segments and entire ORF sequences. There were seven detection methods applied for RDP4 software, involving RDP (Martin and Rybicki, 2000), Chimaera (Posada and Crandall, 2001), BOOTSCAN (Martin et al., 2005), 3Seq (Boni et al., 2007), GENECONV (Padidam et al., 1999), MaxChi (Smith, 1992), and SisScan (Gibbs et al., 2000). When the *p*-value was <0.05 by more than six detection methods, the potential recombination event was considered significant.

### Base Situation Analysis and Evolutionary Dynamic Analysis of Segments

We then calculated the base substitution rate, codon usage frequency, genetic distance, and base substitution saturation analysis of selected sequences for evolutionary dynamic analysis by MEGA X software (Kumar et al., 2018). After importing alignment and setting models, a BEAST XML file was generated via BEAuti v1.10.4 and run by BEAST (Suchard et al., 2018). Combinations of molecular clock type and corresponding relaxed distribution in BEAuti v1.10.4 were variable, thereby influencing the effectiveness of analysis. Thus, we compared the edge likelihood estimation (MLE) values of different model combinations by the path sampling/stepping stone method (PS/SS method) to select the optimal model. Appropriate number of the Markov Chain Monte Carlo (MCMC) chain length and Echo state to screen option were set with a default burn-in of 10%. We browsed the BEAST results via Tracer software, and a value of 200 was considered a reasonable effective sample size (ESS). Information about mutation changes over time was estimated and visualized using the Bayesian skyline plot.

### Ethics Statement

This study used an already-existing collection of data. Samples of patients and rodents isolated in Guangzhou from 2012 to 2016 had been collected based on scrub typhus and rodent-borne disease surveillance data from Guangzhou center for disease control and prevention. We tested the segment sequences and the entire ORF of *TSA56* gene of *O. tsutsugamushi*. All samples used in this study were anonymous. The Institutional Review Board (IRB) of our project is Guangzhou center for disease control and prevention ethics committee.

After reviewing the detail of our project, the Guangzhou center for disease control and prevention ethics committee agreed the researchers to use the data and conduct the project for its in line with the declaration of Helsinki and the “involved biomedical research ethics review approach” (National Health and Family Planning Commission of the People's Republic of China Order No. 11).

## Results

### Data Information

A total of 906 specimens of scrub typhus patients and 65 rodent samples were collected from 2012 to 2016 in Guangzhou, of which 220 patient specimens and 9 rodent samples were tested positive by nested PCR. Among them, 20 patient samples had errors in the sequencing results, for example, the codon could not correctly translate protein, and thus were excluded. Finally, 209 *TSA56* gene segments of sample strains of *O. tsutsugamushi* (200 human specimens and 9 rodent samples) were included in the analysis. And information of 209 sample strains was collected in Supplementary Table 2. Among the strains, 34 strains were amplified with both full sequence and segments. All sample strains have been genotyped by BLAST, with priority given to full sequence genotyping. The lengths of segments and full length sequences were 418–453 and 1,631–1,641 nt, respectively (Supplementary Table 2). The Guangzhou samples were classified into Karp, Kato, Gilliam, TA763, Boryong, and DIVERGENT genotypes.

Reference strains were collected from NCBI. As of July 1, 2019, we searched and selected 326 nucleotide sequences encoding *TSA56* from the sequence databases. These sequences covered at least 80% of ORF. The lengths of reference strains ranged from 1,263 to 1,614 nt. The nucleotide sequences of 209 sample strains and 326 selected strains were aligned and trimmed to the gene segments shared by all strains with MEGA X software (Kumar et al., 2018). In other words, these strains, especially the full-length sequences, were cut into segments as if they were amplified by two pairs of primers, to perform homology analysis and thus to test the authenticity of the segments for genotyping. Strains without isolation year or duplicate sequences were excluded, leaving 139 strains as references to sample strains. We also collected the genotypes of 139 reference strains based on their entire ORF sequences from articles in Pubmed. Other information for the sequences was also retrieved, including the isolation host, isolation year, and isolation location (Supplementary Table 1) (Kim et al., 2017). The reference strains were classified into Karp, Kato, Gilliam, TA763, Boryong, Kawasaki, Shimokoshi, and TA686 genotype.

Sample strains and reference strains in the analysis were represented by “Strain name,” apart from figures. In order to show more information of figures, sample strains in the figures were labeled as follows: “Strain name_Isolated year,” and the symbols of reference strains in the figures were labeled as follows: “Strain name_Isolated region/country_Isolated year.” The reference strains in China named “Isolated region” in their symbols instead of “Isolated country,” such as Zhejiang, Shanxi, or Taiwan.

### Phylogenetic Analysis

**Figure 1:**
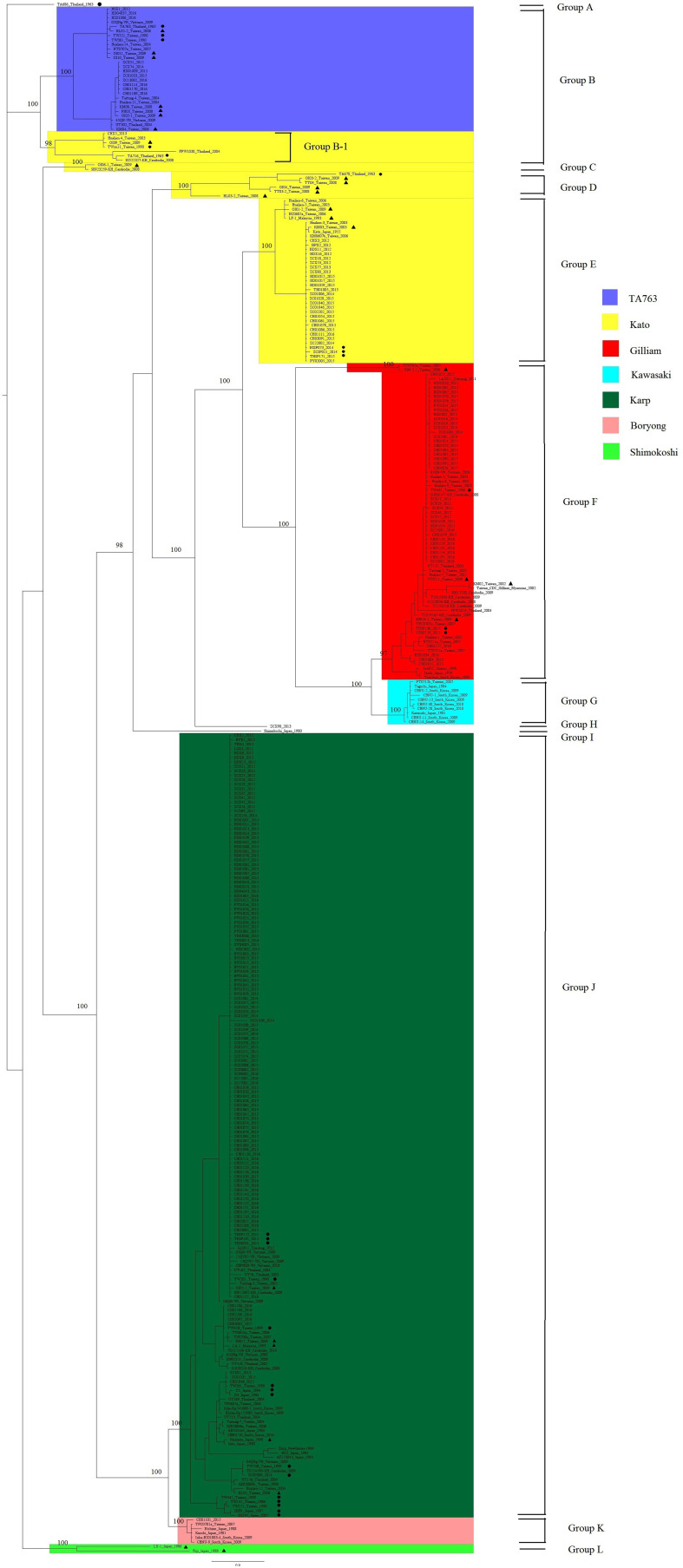
Phylogenetic analysis of 209 sample strains and 139 reference strains. All sequences in figures were labeled specially with information about sequence name, isolated year, and/or isolated region. We selected the GTR + G model which had the smallest BIC, set the MCMC to run 7 million times, and defined the sampling frequency as 1,000 with 25% burn-in. The credibility of the results was checked by determining the average standard deviation of split frequencies and BSRF. Numbers in this figure were probability values over 95% and only the values of main branches were shown due to the limitation of space in this figure. Strains isolated from rodents and chiggers were marked with black dots and black triangles, respectively, to distinguish them from clinically isolated strains of human source. Phylogenetic analysis classified 348 sequences into 12 groups. One branch of Group B was named Group B-1, as showed in the figure. The strains were marked with different colors based on their genotypes, and TA686, one shimokoshi, and a DIVERGENT genotype named ZCX90_2013 were indicated separately.

Phylogenetic analysis was performed using 348 gene segments. We selected the GTR + G model which had the smallest BIC, set the MCMC to run 7 million times, and defined the sampling frequency to be 1,000 with 25% burn-in. The average standard deviation of split frequencies was <0.01, and the potential scale reduction factor of all parameters was close to 1, which represented good credibility. The genetic clusters were defined based on branching node values (probability ≥0.95) (Figure 1). These sequences were mainly classified into 12 groups based on node values, named Group A to Group L. Sequences in the same group were more similar. And we marked segment sequences with different colors to signify their genotypes. And the degree of overlap between 12 groups and color labels can reflect the accuracy of the PCR method, that is, the more overlap, the more accurate the PCR method. By comparing the re-grouping results and the colors of the sequences, we found that the homologous grouping results of segments in Group E–G, J, and K perfectly matched the genotypes of corresponding strains, suggesting that the segment sequences were a good representation of these *TSA56* genotypes. Two independent strains, TA686 and a DIVERGENT strain ZCX90, were also separated in Group A and Group H. The ZCX90 strain was initially classified as a DIVERGENT strain for which genotyping was uncertain, and in this study, it was still separately in Group H. This strain was failed to be re-checked the genotype since it had no entire ORF sequence. But the re-grouping results of Group B–D, I, and L were special. One branch of Group B, named Group B-1 in which strains were all of Kato genotype, showed a closer distance in the phylogenetic tree to TA763 strains in Group B than other Kato strains in Group C–E, suggesting Group B-1 being more homologous to TA763 than Kato genotype,. In other words, segment sequences of Kato strains in Group B-1 were estimated more analogous to corresponding segments of TA763. Besides, Group C and Group D were also of Kato genotype. This suggested that segments of Kato strains contained a wide range of gene differences, and thus some segments had low recognition to Kato. Strain Shimokoshi (Accession No. M63381) in Group I is a well-accepted reference and strains in Group L were also of Shimokoshi genotype. Although all of them were Shimokoshi strains, strains in Group I and Group L were separated in different clusters. We observed that the highest similarity of the entire ORF sequence between two groups was 83% using BLAST, with the segments representing most of the matching sequences (82%). This explained why the two groups belonged to phylogenetically distant groups.

In addition, strains isolated from rodents and chiggers were marked with black dots and black triangles, respectively, to distinguish them from clinically isolated strains of human source (Figure 1). There was no obvious phylogenetical distance between sequences from rodents origin and chigger origin. All sample sequences and main reference sequences from no-human origin fell onto different branches alongside segments of clinical isolates, suggesting the generally low genetic heterogeneity of gene segments from different sources. Group D and L were a separate cluster of Kato and Shimokoshi genotype, respectively, with obviously phylogenetical distance from well-accepted reference strain Kato (Accession no. M63382) in Group E and Shimokoshi (Accession Bo. M63381) in Group I. The difference between Group D, L, and corresponding reference strains was that all segments of Group D, L were from rodents and chiggers. And the whole Group D/L was large phylogenetic distant from their neighbor sequences and thus in a separated cluster.

### Recombination Analysis

#### Recombination Analysis on 24 Strains With Entire ORF Sequences

**Figure 2:**
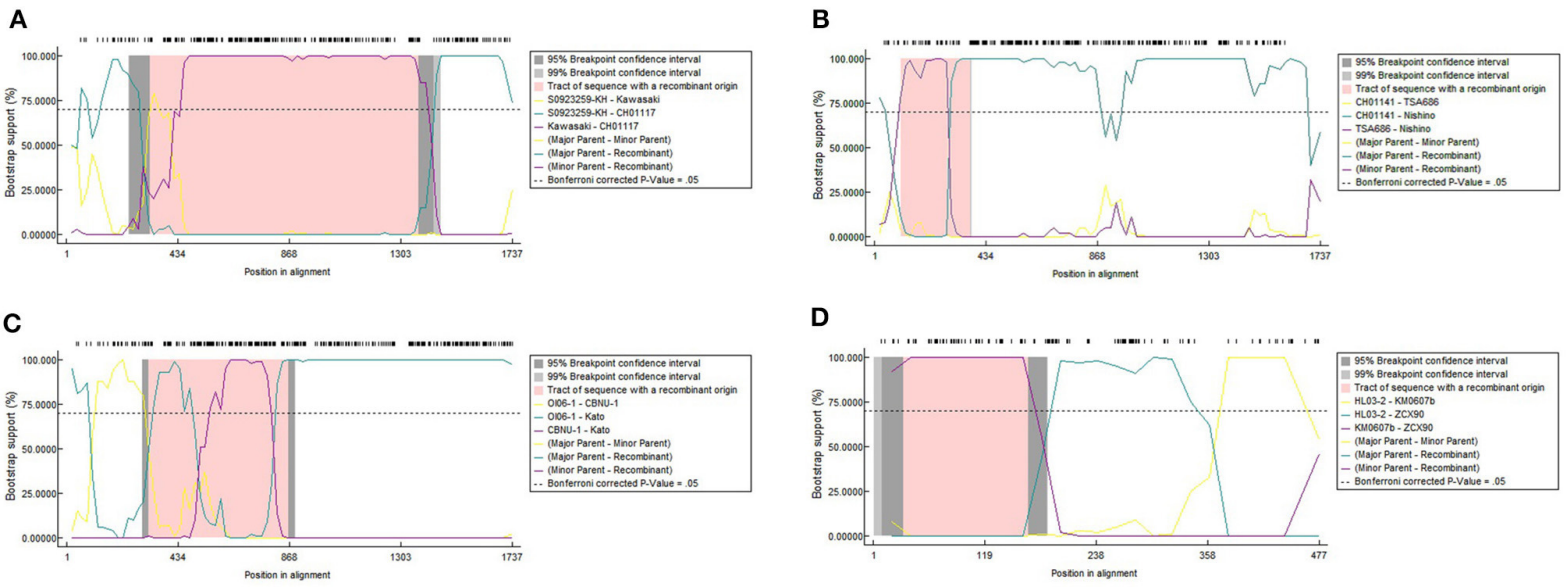
Recombination events of strains with entire ORF sequences **(A–C)** and a recombination event of 348 *TSA56* segments **(D)**. We implemented seven detection methods in RDP4 software to detect the potential recombination events and its corresponding parental sequences. The potential recombination event was considered significant when the *p*-value for more than six detection methods was <0.05 simultaneously. Owing to the limitation of RDP4 software, the symbols of reference strains in this analysis were simplified into “Strain name” instead of “Strain name_Isolated region/country_Isolated year.” **(A–C)** are cross-genotype recombination events with different overlap regions. **(A)** Showed the major overlap occurred between CH01117_2016 and Kawasaki_Japan_1981 revealed by the purple line. Another overlapping region was between CH01117_2016 and S0923259-KH_Cambodia_2008 in green line. The first overlap was wider than the latter one and influenced CH01117_2016 genotype greater. Obvious overlap between different genotypes were shown in **(B)**. Nishino_Japan_1988 (Boryong) had more extensive similarity with Ch01141_2016 (Karp) than TA686_Thailand_1963 (TA686). This similar region contained segments and this might explain why Boryong and Karp genotype were close in Figure 1. In **(C)**, it was detected that Kato_Japan_1955 was partially similar to CBNU-1_South_Korea_2009 and OI06-1_Taiwan_2009 in the segment range, respectively, so the recombination region had two significant overlapping curves. And it seemed that the bootstrapping value of the coincident parts fluctuated obviously. **(D)** Shows one of four potential recombinant products among 348 *TSA56* segment sequences. ZCX90_2013 was of the DIVERGENT genotype. And two inter-segment overlaps were detected between it and Kato strain KM0607b_Taiwan_2006, HL03-2_Taiwan_2008, respectively.

Later we performed a recombination analysis on the strains with entire ORF sequences and researched whether low recognition of some Kato segments was due to recombination specifically between Kato and TA763. We randomly select one sample strain and two reference strains from each group for recombination analysis. One of Group B strains was from Group B-1. Two references aimed to reduce selection bias owing to geographic preference and sequence homology among sample strains. These sequences were required the same as above, that was, with entire ORF sequences. Twenty four strains were chosen (Supplementary Table 3), which only contained five sample sequences because the sample strains were concentrated and had fewer genotypes than the references. The significance threshold for this analysis was set at 70% bootstrapping value (dashed line). In the recombination analysis of multiple genotypes, we observed 8 recombination events involving 10 strains, accounting for 41.67% (10/24) (Table 1). Firstly, no obvious recombination evidence was observed between strains of Group B-1 and other Group strains, and thus it has not yet been proven that weak recognition of Kato segments in Group B-1 was due to recombination. Secondly, recombination evidences were common in entire ORF sequences within the same genotypes and between different genotypes. Comparing the largest recombination region in Table 1, the major overlapping regions of most recombination events occurred between strains of the same genotype, especially Karp and Kato genotype. And cross-genotype overlapping events occurred at different parts of the ORF, involving various genotypes, such as Karp and Boryong/Gilliam/TA763/Kato, Kato and Kawasaki/Gilliam/TA686, Boryong and TA686, and Gilliam and Kawasaki (Figures 2A–C). We also observed that one sequence could have partly similar to several strains of different genotypes at the same time. For example, 05QN_VN (Karp) overlapped with TT0705a (Gilliam), CH01170 (TA763) and S0923259-KH (Kato), respectively, at around 1,300–1,600 nt, even though their bootstrapping values were not close to 100% (Table 1).

**Table 1:** Recombination events with significant evidence for 24 selected sequences.

**No**.	**Recombinant**	**Overlapping region A (without gaps)**	**Strain 1**	**Strain 2**	**Other mainly overlapping region B**	**Strain 1**	**Strain 2**	**The largest region**
1	TT03-2 [Kato]^#^	70–860 nt	TT03-2 [Kato]	TT04 [Kato]	- ^$^	TT03-2 [Kato]	TT0705a [Gilliam]	A
2	CH01117 [Gilliam] (Hualien-1 [Gilliam]^*^)	323–1,233 nt	CH01117 [Gilliam]	Kawasaki [Kawasaki]	–	CH01117 [Gilliam]	S0923259-KH [Kato]	A
3	TT0705a [Gilliam]	282–1,120 nt	05QN-VN [Karp]	TW141 [Karp]	–	TT0705a [Gilliam]	05QN-VN [Karp]	A
4	CH01170 [TA763] (Taitung-4 [TA763])	318–1,025 nt	05QN-VN [Karp]	TW141 [Karp]	–	CH01170 [TA763]	05QN-VN [Karp]	A
5	05QN_VN [Karp]	1,316–1,595 nt	05QN_VN [Karp]	S0923259-KH [Kato]	–	05QN_VN [Karp]	Ch01141 [Karp]	B
6	Kato_Japan [Kato] (HD01017 [Kato])	318–774 nt	Kato_Japan [Kato]	CBNU-1 [Kawasaki]	–	Kato_Japan [Kato]	OI06-1 [Kato]	B
7	OI06-1 [Kato]	21–401 nt	OI06-1 [Kato]	CBNU-1 [Kawasaki]	–	OI06-1 [Kato]	TA686 [TA686]	B
8	Nishino [Boryong]	104–344 nt	Nishino [Boryong]	TA686 [TA686]	–	Nishino [Boryong]	Ch01141 [Karp]	B

# *It is the genotype of the strain in the table*.

* *The strain Hualien-1 in the bracket was with evidence of the same recombination event as the strain CH01117 in that table, suggesting Hualien-1 might harbor recombinant events as well*.

$ *There was no information about the range of other mainly overlapping region*.

Noticeably, the breakpoints of the 24-strain recombination analysis were not fixed. The most common breakpoint was 300 nt, followed by 1,000–1,300, 700–800, and 21–100 nt. But the regions before/after these points, i.e., 318–1,025 nt for CH01170, 70–860 nt for TT03-2 (Table 1) seemed to avoid interrupting the segments we analyzed, that is, the integrity of segments was maintained. To confirm this suppose, we referred to nucleotide sequences and amino acid sequences of special strains of different genotypes (Ohashi et al., 1992; Choi et al., 1999) to infer the VDs and ADs positions of 24 sequences (Supplementary Figure 2A). Interestingly, all major overlapping regions between the same genotype strains contained segments containing VD II-III and AD II. By contrast, except for Nishino (Boryong) and Ch01141 (Karp), CH01117 (Gilliam) and Kawasaki (Kawasaki), Kato_Japan (Kato) and CBNU_1 (Kawasaki), and OI06-1 (Kato) and TA686 (TA686) strain with overlap involving (including containing or interrupting) segment range (Figures 2A,B), the overlap between all different genotype strains was frequently located in the regions containing VD I and AD I and regions containing VD IV and AD IV, accounting for 63.63% (7/11). This suggested segments containing VD II–III and AD II might mainly determine the genotyping. Besides, the overlap between Nishino (Boryong) and Ch01141 (Karp), and CH01117 (Gilliam) and Kawasaki (Kawasaki) respectively explained the close phylogenetical distance of two strains in Figure 1. Another noteworthy finding was about potential inter-segment recombination events. On one hand, segments in all recombination events in the analysis above remained almost stable, with the exception of Kato_Japan (Kato) and HD01017 (Kato). The latte one was with evidence of the same recombination event as the strain Kato_Japan, suggesting HD01017 might harbor recombinant events as well (Table 1). As showed in Figure 2C, Kato_Japan was detected partly similar in the segment range with CBNU-1 (Kawasaki) and OI06-1 (Kato), respectively. But the bootstrapping value of the coincident parts fluctuated obviously.

#### Recombination Analysis on 348 Strain Segments

So we performed another recombination analysis with 348 segments to detect the existence of possible inter-segment recombination events, especially the events related to Kato_Japan (Kato), HD01017 (Kato), CBNU-1 (Kawasaki) and OI06-1 (Kato). Only two significant recombination events were identified in 348-segment analysis (Table 2), which suggested that the segments were almost stable (Table 2). No recombination events related to these four strains were observed, since the analysis rejected them from inter-segment recombinant products by re-checking the bootstrapping value at the segment level. Therefore, even though Kato_Japan (Kato) and HD01017 (Kato) were relatively similar with CBNU-1 and OI06-1, this similarity was not caused by recombination of segments. And we observed some inter-segment recombination events. ZCX90, a strain of DIVERGENT genotype, was detected as a recombinant product with breakpoints at 32 and 160 nt (Figure 2D). Like the above 24 sequences, we compared ZCX90 with several reference strains of different genotypes (Ohashi et al., 1992; Choi et al., 1999) to find location of its VDs and ADs. VD II, VD III, and AD II was at 6–32, 72–91, and 1–61 aa, respectively (Supplementary Figure 2B). So the break point region from around 10–54 aa (32−160 nt) did interrupt VD II and AD II of ZCX90, while VD III was located in the overlap of ZCX90 with HL03-2 (Kato). This may explain the reason for its uncertain genotyping. By contrast, the Kato strains FPW1038, S0522327-KH and TA716 were detected as recombiant strains mainly with another Kato strain Hualien-4. These four strains were all classified into Group B-1. And first three sequences were classified into one branch while the latter one was in another branch of Group B-1. The overlapping region suggested why this four strains were assigned into the same group. And the similarity between Taitung-7 (Karp) and Hualien-4 (Kato) might cause Hualien-4 (Kato) partly different from the three Kato sequences. Besides, since two branches of Group B-1 were closer to TA763 strains than Kato genotypes (Figure 1), it is possible that the overlap between three strains and Hualien-4 may be similar to TA763 segments, although this recombination analysis did not reveal. Besides, the Supplementary re-analysis of recombination on Group B-1 with entire ORF sequences showed that the overlapping region between three strains and Hualien-4 ranged from 426 to 732 nt. With the same method, we verified that this scope included VD II-III and AD II. So the recombination of FPW1038 remained the stability of VD II-III and AD II. Therefore, the region contains VDII and AD II almost remained stable, except for ZCX90. The VD III area was not fixed in series with the region containing VD II and AD II, and VD III may have recombination as revealed in ZCX90. Furthermore, the analysis suggested that some Kato strains were similar with TA763 in the segments but inter-segment recombination events might not be the main reason.

**Table 2:** Recombination events with significant evidence in 348 gene segments.

**No**.	**Recombinant**	**Overlapping region A (without gaps)**	**Strain 1**	**Strain 2**	**Other mainly overlapping region B**	**Strain 1**	**Strain 2**	**The largest region**
1	ZCX90_2013 [DIVERGENT]^#^	32–160 nt	ZCX90_2013 [DIVERGENT]^#^	KM0607b_ Taiwan_2006 [Kato]	- ^$^	ZCX90_2013 [DIVERGENT]	HL03-2_Taiwan_2008 [Kato]	B
2	FPW1038_ Thailand_2004 [Kato] (S0522327-KH_Cambodia_2008 [Kato] TA716_Thailand_1963 [Kato]^*^)	22–246 nt	FPW1038_ Thailand_2004 [Kato]	Hualien-4_Taiwan_2003 [Kato]	–	Taitung_7_ Taiwan_2004 [Karp]	Hualien-4_Taiwan_2003 [Kato]	B

# *It is the genotype of the strain in the table*.

* *The strain S0522327-KH and TA716 in the bracket were with evidence of the same recombination event as the strain FPW1038 in that table, suggesting S0522327-KH and TA716 might harbor recombinant events as well*.

$ *There was no information about the range of other mainly overlapping region*.

One analysis above has verified that entire ORF sequence of *TSA56* was full of potential recombination events. But similarity in segments between TA763 strains and some Kato strains leading to weak recognition might be not caused by inter-segment recombination. Genotyping of Shimokoshi and some Kato strains needed to take into account entire ORF sequences. More importantly, this nest PCR method with special primers is perfectly reliable on genotyping of other strains, including Karp, Boryong, Gilliam, TA763, Kawasaki and part of Kato genotype. This stability is not limited by region and time, because the strains identified come from all over the world and the isolation time spans 1943–2016. And two recombination analysis also verified that the segments, especially regions containing VDII and AD II, were almost stable and intact. Genetic recombination was less important for genetic diversification in these gene segments. Therefore, although this nest PCR method has some uncertainty in determining Shimokoshi and distinguishing some specific Kato strains from TA763, this method still meets the detection need for *O. tsutsugamushi* when considering its cost savings and accurateness. Considering the reliable representation of the segment, we supposed that this segment was a great recognizable part than other regions of *TSA56* ORF. Therefore, we planned to further analyze this representative segment at the molecular level for the dynamic characteristics of *TSA56*. We chose 344 segment sequences, including 208 sample strains and 136 reference strains to perform the following analysis. The analysis about the entire ORF sequences was rejected and 4 potential recombinant sequences were excluded in later analysis, since recombination events can affect the accurateness of evolutionary dynamics analysis. Entire ORF sequence was full of recombination events and recombinants were estimated with recombination disrupting VD II. Point mutations can be inherited via splitting events and recombination events, increasing genetic variations and overestimating point mutation frequencies. Moreover, recombination events between branches from different nodes (e.g., from different genotypes) can generate incompatible sites and influence estimates of tree topology and branch lengths (Salemi and Vandamme, 2009).

### Sequence Composition Analysis

First step was to analyze the elementary composition of the sequences. The average length of amino acid segment sequences (including gaps) was 427 bp (base position). The C + G content of the first position in the codon was high, whereas the A + T contents of the second and third positions were high. The third site had the highest value for T (54.24%) and the lowest value for C (6.03%), suggesting frequency preference of base usage of these segment sequences. This preference further impacted codon usage. The three most commonly used codons were GCU (11.50%), AAU (10.40%), and CCU (10.40%). When the sequences encoded threonine (T), serine (S), and valine (P), they had a preference for ACU, AGU, and CCU, respectively. The sequences had an average of 345 identical pairs (ii), 31 transitional pairs (si), and 40 transversional pairs (sv). The si number was smaller than the sv number, and the *R* was 0.77.

### Evolutionary Dynamic Analysis

The second step was to deduce the dynamic and process of gene evolutionary of *TSA56* through segments, that was, to do evolutionary analysis. Sequence saturation determined whether data needed extra adjustment. So firstly we calculated the pairwise distance (P distance), transitions (TSs), and transversions (TVs) of the sequences, and drew a linear graph with P distance as the horizontal axis and TS/TV as the vertical axis (Supplementary Figure 1). Both TSs and TVs had good linear relationships. As the P distance increased, TSs and TVs had a tendency to increase linearly, reflecting that they had not yet reached saturation. So we could perform evolutionary dynamic analysis on sequences without extra adjustment on the base substitution.

**Figure 3:**
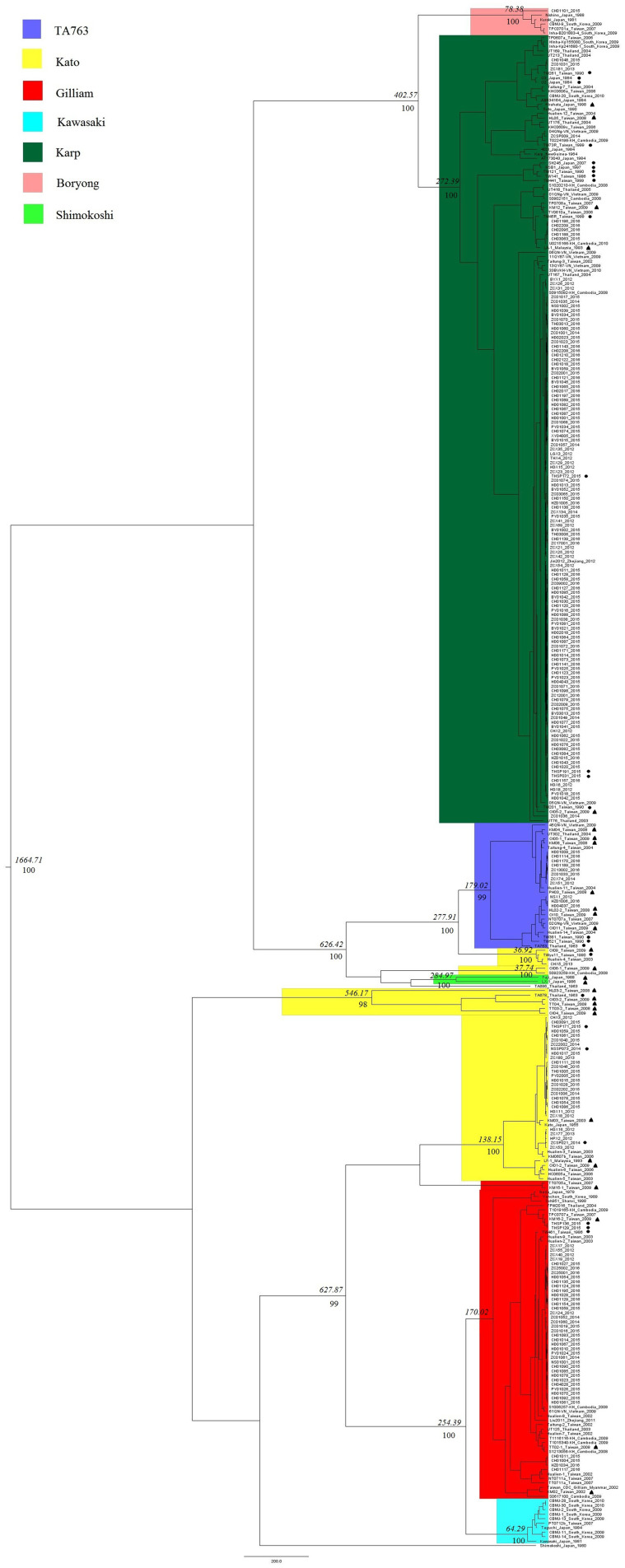
Evolution analysis of *TSA56* segment gene. In BEAuti v1.10.4 software, the MLE value of the UCLD + Exponential model combination was the largest, indicating it as the optimal molecular clock model for evolutionary dynamic analysis. And the Markov Chain Monte Carlo (MCMC) chain length and Echo state to screen option were set to 700 million and 70,000, respectively, with a default burn-in of 10%. Strains isolated from rodents and chiggers were marked with black dots and black triangles, respectively, to distinguish them from clinically isolated strains of human source. Phylodynamic analysis showed that the most recent common ancestor age of segments of the *TSA56* segment gene was 1,664.71 years. Italic numbers represented the node times and the percentiles below the italic numbers were posterior probability values. Since the space in Figure 4 was limited, only values of important branches higher than 0.95 were shown, and these important branches were related to inflection points of genotype differentiation, whereas the values of subsequent branches were omitted.

Evolutionary dynamic analysis showed that the worldwide gene segments of the *TSA56* gene may have originated around 1,664.71 years ago and later evolved and differentiated into two main groups (Figure 3). The Kato genotype appeared earliest, whereas the Kawasaki genotype was the latest. In addition, when all genotypes were compared with each other, we found that Karp and Boryong, Gilliam and Kawasaki, TA763 and Kato had the most recent common parent, revealing that the evolutionary relationship between these two genotypes was relatively close. Among them, the origin of the similarity in segment regions between Group B-1strains and TA763 strains was observed. It was estimated that Group B-1segments was evolved from a most recent common parent shared with TA763 sequences 626 years ago, after excluding the possibility of inter-segment recombination in recombination analysis. And TA686 was identified having a close evolution relationship with OI06-1, which might accounted for the reason of similarity between two corresponding strains in 24-strain recombination analysis. By contrast, Group C/D strains and Group L strains marked in Figure 1 had a far evolution relationship with the Kato strains of Group E and the Shimokoshi strain of Group I, respectively. Therefore, in order to perform genotyping more accurately, entire ORF sequence of Group C, D, and L strains should be considered. From the perspective of geography, the evolutionary relationship showed that the strains of the same regions or countries had a close evolutionary relationship, such as Guangzhou. 97.60% (203/208) of Guangzhou sample strains were located in the closest evolutionary branches with other Guangzhou sample strains, and most were in independent evolutionary branches without reference strains. This percentage of Guangzhou Karp strains which independently located together was 98.40% (123/125). Karp has become a dominant genotype in Guangzhou, because the proportion of Karp genotype in Guangzhou strains has maintained the highest level for several years and the proportion of total Karp strains (125/208) is also the largest. Combined with the high similarity of these stains in Figure 1, we concluded that the gene segments of the Karp genotype stably existed in Guangzhou with high homology, although Karp genotype were previously considered with highly variable (Tao et al., 2018). This suggested the Karp genotype has steadily spread in Guangzhou. On the other hand, the evolutionary relationships showed the geographical preference among strains in geographically close countries. For example, generally the strains nearest with Guangzhou strains were from Taiwan. And strains in Southeast Asian countries are often located on relatively close evolutionary branches.

**Figure 4:**
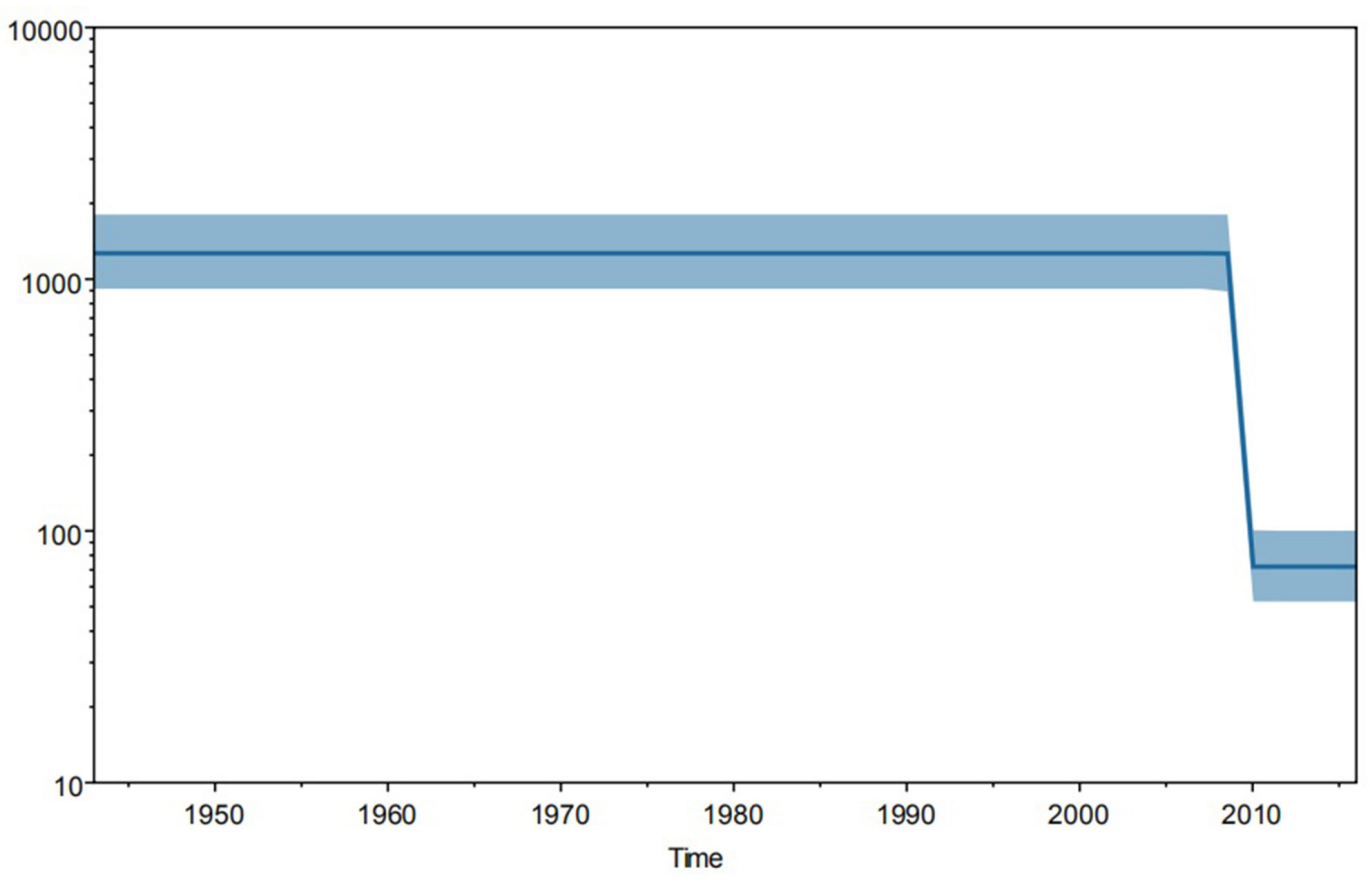
Trend in genetic mutations of 344 strains worldwide from 1943 to 2016. Bayesian skyline plot analysis was performed using Tracer software and demonstrated a Z-shaped trend. Strains before 2009 were global references, whereas strains after 2012 were all from Guangzhou. And the trends before 2009 and after 2012 remained stable. So this shape reflected the stable mutation frequency of global reference strains and Guangzhou sample strains, respectively.

Furthermore, Bayesian skyline plot revealed the trend of genetic mutation in *TSA56* gene segments worldwide (Figure 4). And this mutation trend might reflect the tendency of genetic diversification because of the stability of segments. We observed that this tendency was approximately Z-shaped and remained stable since 1943, with a significant decrease in 2009, and returned to a steady trend from 2010 onwards. So strains since 2010 had a relatively lower mutation frequency than strains isolated before 2009. Among strains since 2010, 4 strains isolated in 2010 came from two countries, while the 211 strains from 2011 to 2016 were all isolated in China, especially Guangzhou (99.05%, 209/211). In more detail, the strains from 2012 to 2016 came from Guangzhou, except for one strain isolated in 2012 in Zhejiang, China. In contrast, strains from 1964 to 2009 were all reference strains. So the trend before 2009 only represented the reference strains while the trend since 2012 only represented the Guangzhou strains. Therefore, this analysis may have a little selection bias on strains. Although the strain bias caused difficulty to estimate the true mutation trend worldwide, two parallel lines indicated that the mutation rates of the references and sample strains remained stable, respectively. And the higher homology of the sample strains might be one of significant reasons which resulted in a lower mutation rate than that of the global reference strains. However, overall epidemic intensity of scrub typhus worldwide was increasing in recent years. Therefore, the significant reason for increased incident rate might not be the change of mutation trend of *TSA56* but other factors. The reason might be that the stable mutation rate did not significantly affect the *TSA56* function.

## Discussion

To date, only genomes of a Boryong (Korea) and a Ikeda (Japan) strain have been completely isolated and assembled (Cho et al., 2007; Nakayama et al., 2008). Analysis of these two genomes showed that more than one third of the genome of which size was about 2.0–2.3 Mb was composed of duplicated genes. The repeats which included conjugative and transposable elements, were caused by amplification of mobile elements, and resulted in intense genome shuffling, making *O. tsutsugamushi* the most repetitive genome of all bacterial species. Due to the high incidence of recombination events, the true phylogeny and the pathogenic mechanism of the *O. tsutsugamushi* are still being studied. In contrast, tremendous information about *TSA56* gene has been uploaded in NCBI, providing a basis for differentiation and pathogenic researches using *TSA56* as a breakthrough point. In this study, the segment contains VD II, VD III, and AD II part of *TSA56* ORF sequence, and the nucleotide length of AD II includes VDII (Supplementary Figure 2). We used the *TSA56* segments to analyze *TSA56* gene mainly by phylogenetic analysis, recombination analysis, and evolutionary dynamic analysis.

The nested PCR method in this study is with two special pairs of primer which amplifies segments instead of entire ORF sequence for disease identification and genotyping. We first proved that the accurateness of this special PCR method and accuracy of segments is not restricted by region and time. The segments could perfectly represent and identify strains of Karp, Boryong, Gilliam, TA763, Kawasaki and part of Kato, while sequence diversification of Shimokoshi and some Kato strains made their genotyping need to consider entire ORF sequences. But this weak recognition of these special Shimokoshi and Kato strains might not be due to recombination. The accurateness and the sensitivity of this detection method to identify and genotype *O. tsutsugamushi* may stem from the uniqueness of VD II and AD II. And Ohashi et al. (1992) proved before that the VD II was most unique in the hydropathic pattern in each TSA protein compared to other VDs, which might partly explain the representation of VD II to *TSA56*.

*TSA56* gene is considered full of point mutations and recombination. As for the recombination mechanism, sexual pili-like cell surface appendages for genes transfer observed in Rickettsia might explain these numerous gene exchanges (Ogata et al., 2006). Gene exchanges between ancestral rickettsiae and other bacteria were probably mediated by conjugation within co-infected amoebae. The *TSA56* gene might undergo recombination between different genotypes through a similar mechanism since co-infection with several genotypes at the same time had been observed both in human hosts (Sonthayanon et al., 2010) and vector mites (Shirai et al., 1982). This study also proved that intragenic recombination was common in the entire *TSA56* ORF gene even among different genotype strains. And our new discovery was that the overlap between different genotypes mainly occurred in VD I and AD I areas, and VD IV and AD IV areas. In contrast, the recombination of segment containing VD II and AD II mainly occurred between the same or homologous genotype strains. The recombination analysis and evolutionary dynamic analysis on segments also verified that diversification of segments was dominated by point mutations instead of inter-segment recombination. But occasional recombination events which interrupted VD II and AD II created new unrecognized genotypes, affecting genotypes classification and increasing genotypes diversification. The ZCX90 was initially identified as a DIVERGENT strain because the highest similarity tested by BLAST was 84% in strain HL03_2 (Accession No. GU120143), and this percentage was low. The uniqueness of ZCX90 was also indirectly proved by strain 47 QNg (Accession No. KJ742341) which was isolated from Vietnam and submitted to NCBI later. 47 QNg showed 99% similarity to ZCX90 at segment level. And in Wongprompitak et al. study, this 47 QNg strain was excluded from Karp, Kato, Boryong, and Gilliam genotypes both at the nucleotide and amino acid levels and classified alone (Wongprompitak et al., 2015). We further observed a recombination event in the ZCX90 sample strain, and the suspected major parent strain was named HL03_2 in this study, which is HL03_2 described above (Accession No. GU120143). This explained the potential reason for emergence of a uncertain genotype. And it provides new insights into gene classification for this detection method, that is, the appearance of new genotypes at the segment level may stem from recombination instead of long-term evolution. Therefore, the genotyping of strains will require more careful and comprehensive consideration for potential new strains. Hosts like chiggers and rodents may be potential influence factors for genes transfer. Groups D and L have only strains from non-human sources, like chiggers and rodents. The sequences of these two groups existed independently, with few recombination events with gene segments of other groups, while other isolates from non-human sources distributed together with human-derived strains. The reason for particularity of Group D and L is not clear. It may be due to the special gene which co-infected with *O. tsutsugamushi* in chiggers and rodents. And this assumption is yet to be verified.

One importance of genotyping study is to understand the evolution and differentiation of *TSA56* ORF sequence. The low recombination frequency and the stable mutation trend mutation for 53 years indicated that the trend of genetic diversity of ORF segment is stable. This segment is a relatively conservative part of the *TSA56* ORF and may anchor and represent the entire *TSA56* ORF gene. And we excluded the change of genetic diversity trend of ORF segment from one potential reason for increased incident rate. Another benefit for this study is to better prepare for studying the relationship between genotypes and treatment, immunity. Currently it has been found that the virulence of different genotypes is different, but the mechanism between the *TSA56* genotypes and bacterial virulence remains to be studied. Ohashi et al. (1992) considered that TSA gene was proposed relating to rickettsial pathogenesis based on the antigenical difference, but the critical sequences of TSA primary structures explaining the virulence between virulent strains (Gilliam, Karp, and Kato) and the avirulent strains (Kawasaki, Kuroki, and Shimokoshi) had not been observed. Nevertheless, Nakayama et al. (2010) believed that virulence might be related to core gene instead of TSA. In his study, the mouse high-virulence and mouse low-virulence strains were classified into same clusters, respectively, in the phylogenetic structure using 11 housekeeping genes among the core gene set of five Rickettsia species. Although the virulence truth still needs the further analysis, the *TSA56* is a useful target to inhibit bacterial virulence. As for the biological function of *TSA56* protein, it is required for *O. tsutsugamushi* adhesion by binding to fibronectin and facilitating bacterial invasion of mammalian host cells (Lin et al., 2012). So successful invasion gives bacteria chances to exert virulence, while inhibiting this protein can prevent *O. tsutsugamushi* from invading cells, no matter what virulence it is. Moreover, before the mechanism is discovered, inductive clinical experience indicated that bacterial genotyping may be useful for treatment. The distinctive symptom of Gilliam and Kato genotype cases were continuous fever and splenomegaly, respectively. And the laboratory features of these two genotype cases were different (Wei et al., 2017). Besides, the response to antibiotic treatment in the Karp cluster was significantly slower than Boryong cluster by comparing the average fever clearance time (Kim et al., 2011). So when the genotypes is known, the patients may get more targeted treatment from clinicians. As for prevention, corresponding immune response to genotype strains is related to better immunity, that is, homotypic immunity.

The possible further development direction of this segment is as a potential part of effective vaccines, especially against Karp, Kato, and Boryong strains. In Choi et al. (1999) study, aa 131–201 from Karp, Kato, and Boryong strains which covered VD II and AD II could react with both homotypic and heterotypic antibodies from these three genotypes, and the aa from Gilliam only had homotypic reaction. And segments containing VD II and AD II have been proved to be a relatively conservative functional part corresponding to their genotypes. In contrast, the region containing VDI and ADI, and region containing VDIII and ADIII failed to have such wide range of antibody responses to *TSA56*. The region containing VD IV and AD IV had different preference for strains, showing cross-reaction among strains Gilliam, Karp, and Boryong but only homotypic reaction to Kato. These cross-reactions of antibodies might be partly contributed by the similarity among ADs in relating regions. Another advantage of this segment is to ease vaccine development. The antigenicity of the AD II region might be largely due to the linear epitopes, because the similarity conclusions of homology analysis and cross-reactivity experiments were based on the primary amino acid sequences. This needs further verification for its structure. However, antigen heterogeneity and differences in the geographical distribution of genotypes hindered the development and improvement of accurate diagnostic methods and effective vaccines (Paris et al., 2013). From the perspective of biological function, it is suspected that point mutations and intragenic recombination, particularly cross-genotype changes, can affect the expression of type-specific antigens, potentially resulting in antigen heterogeneity and immune escape of surface antigens. Antigen heterogeneity is related with vaccine persistence and re-infection situation. Immunity resulting from challenge with homologous strains vaccines will persist at least 1–3 years, whereas immunity resulting from the heterologous strains vaccines is transient, i.e., as short as 1 month (Kelly et al., 2009). Thus, it is possible for re-infection to occur. But the optimal combination of different genotype strains of multivalent vaccines that may be produced is still unclear. On the other hand, the geographical preference of the evolutionary relationship of strains was proved above. Similar strains with only several bases difference occurred in different countries. One hypothesis about various antigenic types distribution of the rickettsiae was due to the species of vector mites. Previously most rickettsiae isolated from patients in the southwestern Japan and northeastern Japan, where the major vector in the endemic area were different, were classified into different virulent groups, Kawasaki/Kuroki strains, and Gilliam/Karp strains, respectively (Ohashi et al., 1992). Furthermore, factors such as temperature, precipitation, and host environment also affected the vectors in to directly influence the transmission of *O. tsutsugamushi* (Peng et al., 2016). Another speculation was about the geographic spread of mites. The mites themselves have poor mobility and mainly relay on rodents to spread to other places. International trade can assist in their geographical spread, such as inadvertently spreading rodents to other countries through cargo transport. Alternatively, the migration of hosts infected by chigger mites could contribute to the spread of the disease. Kuo et al. confirmed the possibility that birds may act as mechanical carriers of chigger mites during pathogen transmission, even between countries (Kuo et al., 2017). More evidence is required to elucidate the close evolutionary relationships of *O. tsutsugamushi* in these geographically distant countries, such as further studies on flyway migratory bird routes and specific bird species.

Some parts in this study still need improvement. The entire ORF sequence of ZCX90 is missing. All sample strains collected from humans serum and rodents spleen lacked information about the vectors. We cannot further verify whether human behavior and environmental factors such as temperature, vector species in Guangzhou are consistent with the transmission of this genotype. And isolated time of both sample strains and reference strains limited the analysis of true mutation trend. What we analyzed were built on the molecular level, while the reaction of amino acids transcribed from the segment to antibodies is unclear. And whether the immune effect of the primary structure of these amino acids is close to the original effect of ADII in *TSA56* remains to be determined. For future researches, more analysis of the tertiary structure of *TSA56* protein, and the immune strength and range of the segment are required to promote vaccine development.

## Data Availability Statement

All datasets generated for this study are included in the article/Supplementary Material.

## Ethics Statement

The studies involving human participants were reviewed and approved by Guangzhou center for disease control and prevention ethics committee. Written informed consent for participation was not required for this study in accordance with the national legislation and the institutional requirements. The animal study was reviewed and approved by Guangzhou center for disease control and prevention ethics committee.

## Author Contributions

JL and YW analyzed the data and wrote the manuscript. XT and PH performed the PCR experiments. JX assisted the analysis. XW and WZ reviewed the manuscript. KC and ZY conceived the study. All authors contributed to the article and approved the submitted version.

## Conflict of Interest

The authors declare that the research was conducted in the absence of any commercial or financial relationships that could be construed as a potential conflict of interest.

## Supplementary Material

Supplementary Figure 1 Saturation analysis of base substitutions in 344 segment sequences. The scatter plot was established with the P distance as the abscissa and TSs and TVs as the ordinates. Both TSs and TVs tended to increase linearly, suggesting sequence saturation of segments have not yet reached saturation. Before a P distance of 0.20, TSs and TVs tended to be similar; however, at larger P distances, TVs gradually exceeded TSs.

Supplementary Figure 2 The position and length of VDs and ADs in a entire ORF sequence **(a)** and a segment **(b)**. **(a)** Represented a Boryong strain Nishino_Japan_1988 (Boryong). The total number of aa of it was 433 aa. **(b)** Revealed the nucleotide structure of ZCX90_2013. ZCX90_2013 contains integral VD II and VD III. The aa of the ZCX90_2013 segment is transcribed from I-S-I-A-D, and the reference strains started from M-S-P-I-S-I-A-D, which meaned that the ZCX90_2013 segment might lack 3 aa at the beginning. Besides, VDIII of ZCX90_2013 was incomplete and part of VDIII was lacking by comparing with references.

Supplementary Table 1 Primers used for nested PCR and DNA segment length of the *TSA56* gene of *O.tsutsugamushi*.

Supplementary Table 2 Information of 209 Guangzhou sample strains and 139 global reference strains.

Supplementary Table 3 Information of 24 selected sequences with entire ORF of *TSA56* gene.
