# Supplementary figures and images for: Representative Genotyping, Recombination and Evolutionary Dynamics Analysis of TSA56 Gene Segment of Orientia tsutsugamushi
Source: Front Cell Infect Microbiol. 2020 Aug 5;10:383. doi: 10.3389/fcimb.2020.00383 (PMC7438794; doi:10.3389/fcimb.2020.00383)

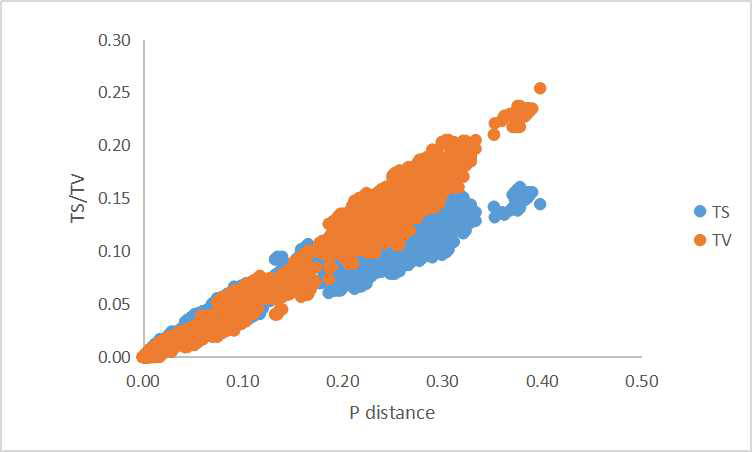

Supplement: Supplementary Figure 1 — Saturation analysis of base substitutions in 344 segment sequences. The scatter plot was established with the P distance as the abscissa and TSs and TVs as the ordinates. Both TSs and TVs tended to increase linearly, suggesting sequence saturation of segments have not yet reached saturation. Before a P distance of 0.20, TSs and TVs tended to be similar; however, at larger P distances, TVs gradually exceeded TSs. [file Data_Sheet_1.ZIP › Supplementary figure/Supplementary figure 1 Saturation analysis.jpg]

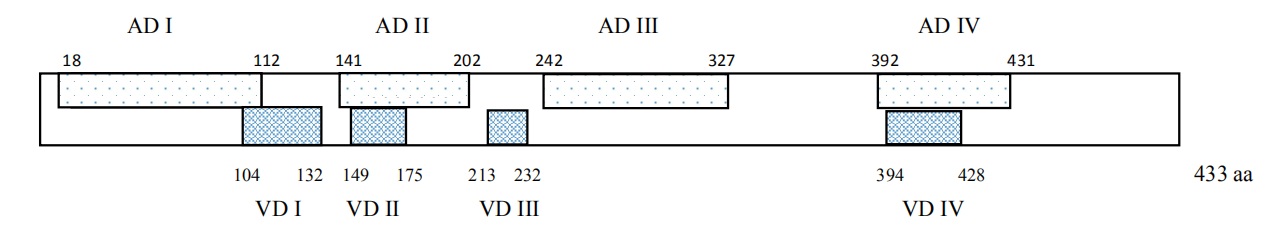

Supplement: Supplementary Figure 1 — Saturation analysis of base substitutions in 344 segment sequences. The scatter plot was established with the P distance as the abscissa and TSs and TVs as the ordinates. Both TSs and TVs tended to increase linearly, suggesting sequence saturation of segments have not yet reached saturation. Before a P distance of 0.20, TSs and TVs tended to be similar; however, at larger P distances, TVs gradually exceeded TSs. [file Data_Sheet_1.ZIP › Supplementary figure/Supplementary figure 2-a.jpg]

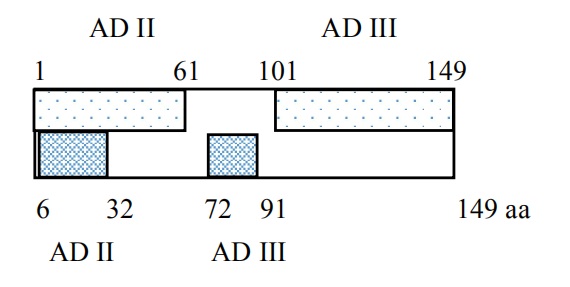

Supplement: Supplementary Figure 1 — Saturation analysis of base substitutions in 344 segment sequences. The scatter plot was established with the P distance as the abscissa and TSs and TVs as the ordinates. Both TSs and TVs tended to increase linearly, suggesting sequence saturation of segments have not yet reached saturation. Before a P distance of 0.20, TSs and TVs tended to be similar; however, at larger P distances, TVs gradually exceeded TSs. [file Data_Sheet_1.ZIP › Supplementary figure/Supplementary figure 2-b.jpg]
