# Supplementary material for: Representative Genotyping, Recombination and Evolutionary Dynamics Analysis of TSA56 Gene Segment of Orientia tsutsugamushi
Source: Front Cell Infect Microbiol. 2020 Aug 5;10:383. doi: 10.3389/fcimb.2020.00383 (PMC7438794; doi:10.3389/fcimb.2020.00383)
Supplement: Supplementary Table 1 — Primers used for nested PCR and DNA segment length of the TSA56 gene of O.tsutsugamushi. [file Data_Sheet_2.docx]

**Supplementary table 1.** Primers used for nested PCR and DNA segment length of the *TSA56* gene of *O.tsutsugamushi*

| **No.** | **Primers** | **Primer sequence (5′→3′)** | **Length of amplified sequence segment** | **Position** |
| --- | --- | --- | --- | --- |
| Pair 1 | tsu-34 | *TCAAGCTTATTGCTAGTGCAATGTCTGC* | 900 – 1100 | 19 – 38 |
|  | tsu-55 | *AGGGATCCCTGCTGCTGTGCTTGCTGCG* |  | 1032 – 1013 |
| Pair 2 | tsu-10 | *GATCAAGCTTCCTCAGCCTACTATAATGCC* | 450 – 500 | 408 – 428 |
|  | tsu-11 | *CTAGGGATCCCGACAGATGCACTATTAGGC* |  | 895 – 876 |
| Pair 3 | ST-A | *TTTCGAACGTGTCTTTAAGC* | 980 – 1100 | (－291) – (－272) |
|  | ST-B | *ACAGATGCACTATTAGGCAA* |  | 893 – 874 |
| Pair 4 | ST-C | *ATGCATATAAACCTAGCGCT* | 960 – 1100 | 764 – 777 |
|  | ST-D | *CTAGAAGTTATAGCGTACACCTGCACTTGC* |  | 1602 – 1580 |

**Supplementary table 2**. Information of 209 Guangzhou sample strains and 139 global reference strains.

| **No.** | **NCBI Accession no.** | **Strain name** | **Isolated location** | **Isolated country** | **Isolated year** | **Isolation host** | **Have the entire ORF sequence(Y or N)** | **Classified before** | **Re-grouping result same as the genotype classified before(Y or N)** |
| --- | --- | --- | --- | --- | --- | --- | --- | --- | --- |
| 1 | MT258708 | BY01002 | Guangzhou, Guangdong, China | China | 2015 | Human | N | Karp | Y |
| 2 | MT258709 | BY01015 | Guangzhou, Guangdong, China | China | 2015 | Human | N | Karp | Y |
| 3 | MT258710 | BY01021 | Guangzhou, Guangdong, China | China | 2015 | Human | N | Karp | Y |
| 4 | MT258711 | BY01034 | Guangzhou, Guangdong, China | China | 2015 | Human | N | Karp | Y |
| 5 | MT258712 | BY01041 | Guangzhou, Guangdong, China | China | 2015 | Human | N | Karp | Y |
| 6 | MT258713 | BY01042 | Guangzhou, Guangdong, China | China | 2015 | Human | N | Karp | Y |
| 7 | MT258714 | BY01045 | Guangzhou, Guangdong, China | China | 2015 | Human | N | Karp | Y |
| 8 | MT258715 | BY01052 | Guangzhou, Guangdong, China | China | 2015 | Human | N | Karp | Y |
| 9 | MT258716 | BY01059 | Guangzhou, Guangdong, China | China | 2015 | Human | N | Karp | Y |
| 10 | MT258717 | BY03013 | Guangzhou, Guangdong, China | China | 2015 | Human | N | Karp | Y |
| 11 | KJ502250 | BYX1 | Guangzhou, Guangdong, China | China | 2012 | Human | N | Karp | Y |
| 12 | MT258718 | CH01018 | Guangzhou, Guangdong, China | China | 2015 | Human | N | Karp | Y |
| 13 | MT258719 | CH01020 | Guangzhou, Guangdong, China | China | 2015 | Human | N | Karp | Y |
| 14 | MT258720 | CH01030 | Guangzhou, Guangdong, China | China | 2016 | Human | N | Karp | Y |
| 15 | MT258721 | CH01043 | Guangzhou, Guangdong, China | China | 2015 | Human | N | Karp | Y |
| 16 | MT258659 | CH01048 | Guangzhou, Guangdong, China | China | 2015 | Human | N | Karp | Y |
| 17 | MT258722 | CH01058 | Guangzhou, Guangdong, China | China | 2015 | Human | N | Karp | Y |
| 18 | MT258723 | CH01064 | Guangzhou, Guangdong, China | China | 2015 | Human | N | Karp | Y |
| 19 | MT258724 | CH01065 | Guangzhou, Guangdong, China | China | 2015 | Human | N | Karp | Y |
| 20 | MT258725 | CH01067 | Guangzhou, Guangdong, China | China | 2015 | Human | N | Karp | Y |
| 21 | MT258726 | CH01073 | Guangzhou, Guangdong, China | China | 2015 | Human | N | Karp | Y |
| 22 | MT258727 | CH01074 | Guangzhou, Guangdong, China | China | 2015 | Human | N | Karp | Y |
| 23 | MT258728 | CH01075 | Guangzhou, Guangdong, China | China | 2015 | Human | N | Karp | Y |
| 24 | MT258729 | CH01079 | Guangzhou, Guangdong, China | China | 2015 | Human | N | Karp | Y |
| 25 | MT258730 | CH01084 | Guangzhou, Guangdong, China | China | 2015 | Human | N | Karp | Y |
| 26 | MT258731 | CH01087 | Guangzhou, Guangdong, China | China | 2015 | Human | N | Karp | Y |
| 27 | MT258732 | CH01089 | Guangzhou, Guangdong, China | China | 2015 | Human | N | Karp | Y |
| 28 | MT258733 | CH01098 | Guangzhou, Guangdong, China | China | 2015 | Human | N | Karp | Y |
| 29 | MT258790 | CH01120 | Guangzhou, Guangdong, China | China | 2016 | Human | Y | Karp | Y |
| 30 | MT258789 | CH01121 | Guangzhou, Guangdong, China | China | 2016 | Human | Y | Karp | Y |
| 31 | MT258817 | CH01128 | Guangzhou, Guangdong, China | China | 2016 | Human | Y | Karp | Y |
| 32 | MT258788 | CH01123 | Guangzhou, Guangdong, China | China | 2016 | Human | Y | Karp | Y |
| 33 | MT258814 | CH01138 | Guangzhou, Guangdong, China | China | 2016 | Human | Y | Karp | Y |
| 34 | MT258813 | CH01139 | Guangzhou, Guangdong, China | China | 2016 | Human | Y | Karp | Y |
| 35 | MT258812 | CH01141 | Guangzhou, Guangdong, China | China | 2016 | Human | Y | Karp | Y |
| 36 | MT258811 | CH01143 | Guangzhou, Guangdong, China | China | 2016 | Human | Y | Karp | Y |
| 37 | MT258810 | CH01150 | Guangzhou, Guangdong, China | China | 2016 | Human | Y | Karp | Y |
| 38 | MT258808 | CH01157 | Guangzhou, Guangdong, China | China | 2016 | Human | Y | Karp | Y |
| 39 | MT258806 | CH01171 | Guangzhou, Guangdong, China | China | 2016 | Human | Y | Karp | Y |
| 40 | MT258818 | CH01127 | Guangzhou, Guangdong, China | China | 2016 | Human | Y | Karp | Y |
| 41 | MT258663 | CH01186 | Guangzhou, Guangdong, China | China | 2016 | Human | N | Karp | Y |
| 42 | MT258805 | CH01188 | Guangzhou, Guangdong, China | China | 2016 | Human | Y | Karp | Y |
| 43 | MT258803 | CH01197 | Guangzhou, Guangdong, China | China | 2016 | Human | Y | Karp | Y |
| 44 | MT258802 | CH01210 | Guangzhou, Guangdong, China | China | 2016 | Human | Y | Karp | Y |
| 45 | MT258734 | CH02017 | Guangzhou, Guangdong, China | China | 2015 | Human | N | Karp | Y |
| 46 | MT258660 | CH02095 | Guangzhou, Guangdong, China | China | 2015 | Human | N | Karp | Y |
| 47 | MT258735 | CH02122 | Guangzhou, Guangdong, China | China | 2016 | Human | N | Karp | Y |
| 48 | MT258801 | CH02208 | Guangzhou, Guangdong, China | China | 2016 | Human | Y | Karp | Y |
| 49 | MT258800 | CH02209 | Guangzhou, Guangdong, China | China | 2016 | Human | Y | Karp | Y |
| 50 | MT258661 | CH03063 | Guangzhou, Guangdong, China | China | 2015 | Human | N | Karp | Y |
| 51 | MT258736 | CH03082 | Guangzhou, Guangdong, China | China | 2015 | Human | N | Karp | Y |
| 52 | KJ502271 | CHX2 | Guangzhou, Guangdong, China | China | 2012 | Human | N | Karp | Y |
| 53 | MT258737 | HD01001 | Guangzhou, Guangdong, China | China | 2015 | Human | N | Karp | Y |
| 54 | MT258738 | HD01011 | Guangzhou, Guangdong, China | China | 2015 | Human | N | Karp | Y |
| 55 | MT258739 | HD01013 | Guangzhou, Guangdong, China | China | 2015 | Human | N | Karp | Y |
| 56 | MT258740 | HD01014 | Guangzhou, Guangdong, China | China | 2015 | Human | N | Karp | Y |
| 57 | MT258741 | HD01039 | Guangzhou, Guangdong, China | China | 2015 | Human | N | Karp | Y |
| 58 | MT258742 | HD01042 | Guangzhou, Guangdong, China | China | 2015 | Human | N | Karp | Y |
| 59 | MT258743 | HD01060 | Guangzhou, Guangdong, China | China | 2015 | Human | N | Karp | Y |
| 60 | MT258744 | HD01062 | Guangzhou, Guangdong, China | China | 2015 | Human | N | Karp | Y |
| 61 | MT258745 | HD01076 | Guangzhou, Guangdong, China | China | 2015 | Human | N | Karp | Y |
| 62 | MT258746 | HD01077 | Guangzhou, Guangdong, China | China | 2015 | Human | N | Karp | Y |
| 63 | MT258797 | HD01082 | Guangzhou, Guangdong, China | China | 2015 | Human | Y | Karp | Y |
| 64 | MT258747 | HD01085 | Guangzhou, Guangdong, China | China | 2015 | Human | N | Karp | Y |
| 65 | MT258748 | HD01087 | Guangzhou, Guangdong, China | China | 2015 | Human | N | Karp | Y |
| 66 | MT258749 | HD01088 | Guangzhou, Guangdong, China | China | 2015 | Human | N | Karp | Y |
| 67 | MT258750 | HD02018 | Guangzhou, Guangdong, China | China | 2015 | Human | N | Karp | Y |
| 68 | MT258751 | HD02023 | Guangzhou, Guangdong, China | China | 2015 | Human | N | Karp | Y |
| 69 | MT258753 | HD04043 | Guangzhou, Guangdong, China | China | 2015 | Human | N | Karp | Y |
| 70 | KJ502269 | HDX6 | Guangzhou, Guangdong, China | China | 2012 | Human | N | Karp | Y |
| 71 | KJ502258 | HDX8 | Guangzhou, Guangdong, China | China | 2012 | Human | N | Karp | Y |
| 72 | KJ502252 | HDX15 | Guangzhou, Guangdong, China | China | 2012 | Human | N | Karp | Y |
| 73 | MT258754 | HZ01005 | Guangzhou, Guangdong, China | China | 2016 | Human | N | Karp | Y |
| 74 | MT258796 | HZ01015 | Guangzhou, Guangdong, China | China | 2016 | Human | Y | Karp | Y |
| 75 | KJ502251 | LGX3 | Guangzhou, Guangdong, China | China | 2012 | Human | N | Karp | Y |
| 76 | MT258756 | NS01002 | Guangzhou, Guangdong, China | China | 2015 | Human | N | Karp | Y |
| 77 | MT258757 | PY01016 | Guangzhou, Guangdong, China | China | 2015 | Human | N | Karp | Y |
| 78 | MT258758 | PY01018 | Guangzhou, Guangdong, China | China | 2015 | Human | N | Karp | Y |
| 79 | MT258759 | PY01023 | Guangzhou, Guangdong, China | China | 2015 | Human | N | Karp | Y |
| 80 | MT258760 | PY01025 | Guangzhou, Guangdong, China | China | 2015 | Human | N | Karp | Y |
| 81 | MT258761 | PY01034 | Guangzhou, Guangdong, China | China | 2015 | Human | N | Karp | Y |
| 82 | MT258762 | PY01035 | Guangzhou, Guangdong, China | China | 2015 | Human | N | Karp | Y |
| 83 | MT258763 | PY01081 | Guangzhou, Guangdong, China | China | 2015 | Human | N | Karp | Y |
| 84 | MT258764 | TH03006 | Guangzhou, Guangdong, China | China | 2015 | Human | N | Karp | Y |
| 85 | MT258765 | TH03013 | Guangzhou, Guangdong, China | China | 2016 | Human | N | Karp | Y |
| 86 | KJ502262 | THX4 | Guangzhou, Guangdong, China | China | 2012 | Human | N | Karp | Y |
| 87 | MT258769 | XY04005 | Guangzhou, Guangdong, China | China | 2015 | Human | N | Karp | Y |
| 88 | MT258770 | ZC01001 | Guangzhou, Guangdong, China | China | 2014 | Human | N | Karp | Y |
| 89 | MT258771 | ZC01017 | Guangzhou, Guangdong, China | China | 2015 | Human | N | Karp | Y |
| 90 | MT258772 | ZC01022 | Guangzhou, Guangdong, China | China | 2015 | Human | N | Karp | Y |
| 91 | MT258773 | ZC01023 | Guangzhou, Guangdong, China | China | 2015 | Human | N | Karp | Y |
| 92 | MT258662 | ZC01031 | Guangzhou, Guangdong, China | China | 2015 | Human | N | Karp | Y |
| 93 | MT258774 | ZC01035 | Guangzhou, Guangdong, China | China | 2014 | Human | N | Karp | Y |
| 94 | MT258652 | ZC01036 | Guangzhou, Guangdong, China | China | 2014 | Human | N | Karp | Y |
| 95 | MT258775 | ZC01038 | Guangzhou, Guangdong, China | China | 2015 | Human | N | Karp | Y |
| 96 | MT258776 | ZC01049 | Guangzhou, Guangdong, China | China | 2014 | Human | N | Karp | Y |
| 97 | MT258777 | ZC01057 | Guangzhou, Guangdong, China | China | 2014 | Human | N | Karp | Y |
| 98 | MT258778 | ZC01068 | Guangzhou, Guangdong, China | China | 2015 | Human | N | Karp | Y |
| 99 | MT258779 | ZC01070 | Guangzhou, Guangdong, China | China | 2015 | Human | N | Karp | Y |
| 100 | MT258780 | ZC01071 | Guangzhou, Guangdong, China | China | 2015 | Human | N | Karp | Y |
| 101 | MT258781 | ZC01072 | Guangzhou, Guangdong, China | China | 2015 | Human | N | Karp | Y |
| 102 | MT258782 | ZC01074 | Guangzhou, Guangdong, China | China | 2015 | Human | N | Karp | Y |
| 103 | MT258783 | ZC02001 | Guangzhou, Guangdong, China | China | 2015 | Human | N | Karp | Y |
| 104 | MT258784 | ZC02008 | Guangzhou, Guangdong, China | China | 2015 | Human | N | Karp | Y |
| 105 | MT258785 | ZC03065 | Guangzhou, Guangdong, China | China | 2015 | Human | N | Karp | Y |
| 106 | MT258786 | ZC09002 | Guangzhou, Guangdong, China | China | 2016 | Human | N | Karp | Y |
| 107 | MT258794 | ZC12001 | Guangzhou, Guangdong, China | China | 2016 | Human | Y | Karp | Y |
| 108 | MT258793 | ZC17001 | Guangzhou, Guangdong, China | China | 2016 | Human | Y | Karp | Y |
| 109 | KJ502260 | ZCX21 | Guangzhou, Guangdong, China | China | 2012 | Human | N | Karp | Y |
| 110 | KJ502267 | ZCX23 | Guangzhou, Guangdong, China | China | 2012 | Human | N | Karp | Y |
| 111 | KJ502254 | ZCX25 | Guangzhou, Guangdong, China | China | 2012 | Human | N | Karp | Y |
| 112 | KJ502243 | ZCX26 | Guangzhou, Guangdong, China | China | 2012 | Human | N | Karp | Y |
| 113 | KJ502265 | ZCX29 | Guangzhou, Guangdong, China | China | 2012 | Human | N | Karp | Y |
| 114 | KJ502261 | ZCX31 | Guangzhou, Guangdong, China | China | 2012 | Human | N | Karp | Y |
| 115 | KJ502241 | ZCX35 | Guangzhou, Guangdong, China | China | 2012 | Human | N | Karp | Y |
| 116 | KJ502248 | ZCX41 | Guangzhou, Guangdong, China | China | 2012 | Human | N | Karp | Y |
| 117 | KJ502255 | ZCX42 | Guangzhou, Guangdong, China | China | 2012 | Human | N | Karp | Y |
| 118 | KJ502239 | ZCX54 | Guangzhou, Guangdong, China | China | 2012 | Human | N | Karp | Y |
| 119 | KJ502249 | ZCX69 | Guangzhou, Guangdong, China | China | 2012 | Human | N | Karp | Y |
| 120 | KJ502253 | ZCX81 | Guangzhou, Guangdong, China | China | 2013 | Human | N | Karp | Y |
| 121 | KJ502238 | ZCX134 | Guangzhou, Guangdong, China | China | 2014 | Human | N | Karp | Y |
| 122 | MT258766 | THSP031 | Guangzhou, Guangdong, China | China | 2015 | Rodent | N | Karp | Y |
| 123 | MT258767 | THSP172 | Guangzhou, Guangdong, China | China | 2015 | Rodent | N | Karp | Y |
| 124 | MT258768 | THSP191 | Guangzhou, Guangdong, China | China | 2015 | Rodent | N | Karp | Y |
| 125 | MT258655 | ZCSP009 | Guangzhou, Guangdong, China | China | 2014 | Rodent | N | Karp | Y |
| 126 | MT258654 | CH01101 | Guangzhou, Guangdong, China | China | 2015 | Human | N | Boryong | Y |
| 127 | MT258681 | CH01004 | Guangzhou, Guangdong, China | China | 2015 | Human | N | Gilliam | Y |
| 128 | MT258682 | CH01011 | Guangzhou, Guangdong, China | China | 2015 | Human | N | Gilliam | Y |
| 129 | MT258683 | CH01014 | Guangzhou, Guangdong, China | China | 2015 | Human | N | Gilliam | Y |
| 130 | MT258684 | CH01023 | Guangzhou, Guangdong, China | China | 2015 | Human | N | Gilliam | Y |
| 131 | MT258820 | CH01027 | Guangzhou, Guangdong, China | China | 2015 | Human | Y | Gilliam | Y |
| 132 | MT258685 | CH01059 | Guangzhou, Guangdong, China | China | 2015 | Human | N | Gilliam | Y |
| 133 | MT258686 | CH01083 | Guangzhou, Guangdong, China | China | 2015 | Human | N | Gilliam | Y |
| 134 | MT258687 | CH01085 | Guangzhou, Guangdong, China | China | 2015 | Human | N | Gilliam | Y |
| 135 | MT258688 | CH01090 | Guangzhou, Guangdong, China | China | 2015 | Human | N | Gilliam | Y |
| 136 | MT258689 | CH01092 | Guangzhou, Guangdong, China | China | 2015 | Human | N | Gilliam | Y |
| 137 | MT258819 | CH01117 | Guangzhou, Guangdong, China | China | 2016 | Human | Y | Gilliam | Y |
| 138 | MT258787 | CH01124 | Guangzhou, Guangdong, China | China | 2016 | Human | Y | Gilliam | Y |
| 139 | MT258816 | CH01129 | Guangzhou, Guangdong, China | China | 2016 | Human | Y | Gilliam | Y |
| 140 | MT258815 | CH01135 | Guangzhou, Guangdong, China | China | 2016 | Human | Y | Gilliam | Y |
| 141 | MT258809 | CH01154 | Guangzhou, Guangdong, China | China | 2016 | Human | Y | Gilliam | Y |
| 142 | MT258804 | CH01195 | Guangzhou, Guangdong, China | China | 2016 | Human | Y | Gilliam | Y |
| 143 | MT258690 | CH04028 | Guangzhou, Guangdong, China | China | 2015 | Human | N | Gilliam | Y |
| 144 | MT258691 | HD01010 | Guangzhou, Guangdong, China | China | 2015 | Human | N | Gilliam | Y |
| 145 | MT258798 | HD01028 | Guangzhou, Guangdong, China | China | 2015 | Human | Y | Gilliam | Y |
| 146 | MT258692 | HD01054 | Guangzhou, Guangdong, China | China | 2015 | Human | N | Gilliam | Y |
| 147 | MT258693 | HD01061 | Guangzhou, Guangdong, China | China | 2015 | Human | N | Gilliam | Y |
| 148 | MT258694 | HD01067 | Guangzhou, Guangdong, China | China | 2015 | Human | N | Gilliam | Y |
| 149 | MT258695 | HD01070 | Guangzhou, Guangdong, China | China | 2015 | Human | N | Gilliam | Y |
| 150 | MT258696 | HD01078 | Guangzhou, Guangdong, China | China | 2015 | Human | N | Gilliam | Y |
| 151 | MT258795 | HZ01034 | Guangzhou, Guangdong, China | China | 2016 | Human | Y | Gilliam | Y |
| 152 | MT258697 | NS01001 | Guangzhou, Guangdong, China | China | 2015 | Human | N | Gilliam | Y |
| 153 | MT258698 | PY01024 | Guangzhou, Guangdong, China | China | 2015 | Human | N | Gilliam | Y |
| 154 | MT258699 | PY01026 | Guangzhou, Guangdong, China | China | 2015 | Human | N | Gilliam | Y |
| 155 | MT258702 | ZC01016 | Guangzhou, Guangdong, China | China | 2015 | Human | N | Gilliam | Y |
| 156 | MT258703 | ZC01019 | Guangzhou, Guangdong, China | China | 2015 | Human | N | Gilliam | Y |
| 157 | MT258704 | ZC01052 | Guangzhou, Guangdong, China | China | 2014 | Human | N | Gilliam | Y |
| 158 | MT258705 | ZC01060 | Guangzhou, Guangdong, China | China | 2014 | Human | N | Gilliam | Y |
| 159 | MT258706 | ZC01061 | Guangzhou, Guangdong, China | China | 2014 | Human | N | Gilliam | Y |
| 160 | MT258707 | ZC25001 | Guangzhou, Guangdong, China | China | 2016 | Human | N | Gilliam | Y |
| 161 | MT258791 | ZC25002 | Guangzhou, Guangdong, China | China | 2016 | Human | Y | Gilliam | Y |
| 162 | KJ502242 | ZCX17 | Guangzhou, Guangdong, China | China | 2012 | Human | N | Gilliam | Y |
| 163 | KJ502272 | ZCX19 | Guangzhou, Guangdong, China | China | 2012 | Human | N | Gilliam | Y |
| 164 | KJ502264 | ZCX24 | Guangzhou, Guangdong, China | China | 2012 | Human | N | Gilliam | Y |
| 165 | KJ502247 | ZCX40 | Guangzhou, Guangdong, China | China | 2012 | Human | N | Gilliam | Y |
| 166 | KJ502246 | ZCX55 | Guangzhou, Guangdong, China | China | 2012 | Human | N | Gilliam | Y |
| 167 | MT258700 | THSP129 | Guangzhou, Guangdong, China | China | 2015 | Rodent | N | Gilliam | Y |
| 168 | MT258701 | THSP136 | Guangzhou, Guangdong, China | China | 2015 | Rodent | N | Gilliam | Y |
| 169 | MT258653 | CH01114 | Guangzhou, Guangdong, China | China | 2016 | Human | N | TA763 | Y |
| 170 | MT258807 | CH01170 | Guangzhou, Guangdong, China | China | 2016 | Human | Y | TA763 | Y |
| 171 | MT258658 | CH01189 | Guangzhou, Guangdong, China | China | 2016 | Human | N | TA763 | Y |
| 172 | MT258656 | HD01009 | Guangzhou, Guangdong, China | China | 2016 | Human | N | TA763 | Y |
| 173 | MT258752 | HD04037 | Guangzhou, Guangdong, China | China | 2016 | Human | N | TA763 | Y |
| 174 | MT258755 | HZ01006 | Guangzhou, Guangdong, China | China | 2016 | Human | Y | TA763 | Y |
| 175 | KJ502270 | NSX1 | Guangzhou, Guangdong, China | China | 2012 | Human | N | TA763 | Y |
| 176 | MT258657 | ZC01033 | Guangzhou, Guangdong, China | China | 2015 | Human | N | TA763 | Y |
| 177 | MT258821 | ZC13002 | Guangzhou, Guangdong, China | China | 2016 | Human | N | TA763 | Y |
| 178 | KJ502263 | ZCX51 | Guangzhou, Guangdong, China | China | 2012 | Human | N | TA763 | Y |
| 179 | KJ502268 | ZCX74 | Guangzhou, Guangdong, China | China | 2014 | Human | N | TA763 | Y |
| 180 | KJ502244 | CHX5 | Guangzhou, Guangdong, China | China | 2013 | Human | N | Kato | classified in Group A with TA763 |
| 181 | MT258664 | CH01054 | Guangzhou, Guangdong, China | China | 2015 | Human | N | Kato | Y |
| 182 | MT258665 | CH01061 | Guangzhou, Guangdong, China | China | 2015 | Human | N | Kato | Y |
| 183 | MT258666 | CH01078 | Guangzhou, Guangdong, China | China | 2015 | Human | N | Kato | Y |
| 184 | MT258667 | CH01086 | Guangzhou, Guangdong, China | China | 2015 | Human | N | Kato | Y |
| 185 | MT258668 | CH01111 | Guangzhou, Guangdong, China | China | 2016 | Human | N | Kato | Y |
| 186 | MT258669 | CH03091 | Guangzhou, Guangdong, China | China | 2015 | Human | N | Kato | Y |
| 187 | KJ502245 | CHX3 | Guangzhou, Guangdong, China | China | 2012 | Human | N | Kato | Y |
| 188 | MT258670 | HD01015 | Guangzhou, Guangdong, China | China | 2015 | Human | N | Kato | Y |
| 189 | MT258799 | HD01017 | Guangzhou, Guangdong, China | China | 2015 | Human | Y | Kato | Y |
| 190 | MT258671 | HD01059 | Guangzhou, Guangdong, China | China | 2015 | Human | N | Kato | Y |
| 191 | KJ502266 | HDX11 | Guangzhou, Guangdong, China | China | 2012 | Human | N | Kato | Y |
| 192 | KJ502257 | HDX16 | Guangzhou, Guangdong, China | China | 2012 | Human | N | Kato | Y |
| 193 | KJ502259 | HPX2 | Guangzhou, Guangdong, China | China | 2012 | Human | N | Kato | Y |
| 194 | MT258673 | PY02005 | Guangzhou, Guangdong, China | China | 2015 | Human | N | Kato | Y |
| 195 | MT258651 | TH01005 | Guangzhou, Guangdong, China | China | 2015 | Human | N | Kato | Y |
| 196 | MT258675 | ZC01006 | Guangzhou, Guangdong, China | China | 2014 | Human | N | Kato | Y |
| 197 | MT258676 | ZC01028 | Guangzhou, Guangdong, China | China | 2015 | Human | N | Kato | Y |
| 198 | MT258677 | ZC01040 | Guangzhou, Guangdong, China | China | 2015 | Human | N | Kato | Y |
| 199 | MT258678 | ZC01046 | Guangzhou, Guangdong, China | China | 2015 | Human | N | Kato | Y |
| 200 | MT258679 | ZC02202 | Guangzhou, Guangdong, China | China | 2015 | Human | N | Kato | Y |
| 201 | MT258792 | ZC22002 | Guangzhou, Guangdong, China | China | 2016 | Human | Y | Kato | Y |
| 202 | KJ502240 | ZCX18 | Guangzhou, Guangdong, China | China | 2012 | Human | N | Kato | Y |
| 203 | KJ502256 | ZCX53 | Guangzhou, Guangdong, China | China | 2012 | Human | N | Kato | Y |
| 204 | KJ502274 | ZCX77 | Guangzhou, Guangdong, China | China | 2013 | Human | N | Kato | Y |
| 205 | KJ502273 | ZCX80 | Guangzhou, Guangdong, China | China | 2013 | Human | N | Kato | Y |
| 206 | MT258672 | NSSP073 | Guangzhou, Guangdong, China | China | 2014 | Rodent | N | Kato | Y |
| 207 | MT258674 | THSP171 | Guangzhou, Guangdong, China | China | 2015 | Rodent | N | Kato | Y |
| 208 | MT258680 | ZCSP021 | Guangzhou, Guangdong, China | China | 2014 | Rodent | N | Kato | Y |
| 209 | KJ502275 | ZCX90 | Guangzhou, Guangdong, China | China | 2013 | Human | N | DIVERGENT | Y |
| 210 | AB534164 | Matsuzawa | Niigata, Japan | Japan | 1984 | Human | Y | Karp | Y |
| 211 | JQ898367 | CBNU-20 | South Korea | South Korea | 2010 | Human | Y | Karp | Y |
| 212 | AF173043 | Matsuzawa | Niigata, Japan | Japan | 1984 | Human | Y | Karp | Y |
| 213 | AF173047 | 402I | Niigata, Japan | Japan | 1984 | Human | Y | Karp | Y |
| 214 | AF201835 | Hirahata | Obara, Aichi, Japan | Japan | 1998 | Chigger | Y | Karp | Y |
| 215 | JX235718 | Sato | Fukushima, Japan | Japan | 1990 | Human | Y | Karp | Y |
| 216 | AY222636 | TW261 | Lan-Yu, Taiwan, China | China | 1990 | Rodent | Y | Karp | Y |
| 217 | EF213088 | UT213 | SangKhom, UdornThani, Thailand | Thailand | 2004 | Human | Y | Karp | Y |
| 218 | EF213092 | UT169 | Muang, UdornThani, Thailand | Thailand | 2004 | Human | Y | Karp | Y |
| 219 | EU551148 | Taitung-7 | Taitung, Taiwan, China | China | 2004 | Human | Y | Karp | Y |
| 220 | GQ332744 | TP0607a | Taipei, Taiwan, China | China | 2006 | Human | Y | Karp | Y |
| 221 | GQ332748 | KHC0606a | Kaohsiung, Taiwan, China | China | 2006 | Human | Y | Karp | Y |
| 222 | HQ660203 | Inha-Kp241680-1 | South Korea | South Korea | 2009 | Human | Y | Karp | Y |
| 223 | HQ660207 | Inha-Kp155080 | South Korea | South Korea | 2009 | Human | Y | Karp | Y |
| 224 | KC688322 | O2 | Fukushima, Japan | Japan | 1984 | Rodent | Y | Karp | Y |
| 225 | KC688323 | O3 | Tokushima, Japan | Japan | 1984 | Rodent | Y | Karp | Y |
| 226 | M33004 | Karp | New Guinea | New Guinea | 1943 | Human | Y | Karp | Y |
| 227 | DQ323176 | Hualien-12 | Hualien, Taiwan, China | China | 2004 | Human | Y | Karp | Y |
| 228 | EF213081 | UT176 | BanPhu, UdornThani, Thailand | Thailand | 2004 | Human | Y | Karp | Y |
| 229 | GQ332747 | KHC0609c | Kaohsiung, Taiwan, China | China | 2006 | Human | Y | Karp | Y |
| 230 | GU120145 | HL05 | Hualien, Taiwan, China | China | 2008 | Chigger | Y | Karp | Y |
| 231 | HQ718450 | 04QNg_VN | QuangNgai, Vietnam | Vietnam | 2009 | Human | Y | Karp | Y |
| 232 | AF173049 | LA-1 | Malaysia | Malaysia | 1993 | Chigger | Y | Karp | Y |
| 233 | HQ718432 | T0224198_KH | Kandal, Cambodia | Cambodia | 2009 | Human | Y | Karp | Y |
| 234 | AY222628 | TW73R | Kinmen, Taiwan, China | China | 1999 | Rodent | Y | Karp | Y |
| 235 | AY222632 | TW45R | Kinmen, Taiwan, China | China | 1999 | Rodent | Y | Karp | Y |
| 236 | AY222637 | TW201 | Lan-Yu, Taiwan, China | China | 1990 | Rodent | Y | Karp | Y |
| 237 | AY357216 | Taitung-3 | Taiwan, China | China | 2002 | Human | Y | Karp | Y |
| 238 | EF213078 | UT76 | Muang, UdornThani, Thailand | Thailand | 2003 | Human | Y | Karp | Y |
| 239 | EF213080 | UT167 | Phen, UdornThani, Thailand | Thailand | 2004 | Human | Y | Karp | Y |
| 240 | EF213090 | UT418 | Muang, UdornThani, Thailand | Thailand | 2005 | Human | Y | Karp | Y |
| 241 | GQ332743 | TY0610a | Taoyuan, Taiwan, China | China | 2006 | Human | Y | Karp | Y |
| 242 | GQ332745 | TP0708a | Taipei, Taiwan, China | China | 2007 | Human | Y | Karp | Y |
| 243 | GU446592 | KM12 | Kinmen, Taiwan, China | China | 2009 | Chigger | Y | Karp | Y |
| 244 | GU446614 | OI05-2 | Lan-Yu, Taiwan, China | China | 2009 | Chigger | Y | Karp | Y |
| 245 | HQ718422 | S0902151_KH | Kratie, Cambodia | Cambodia | 2008 | Human | Y | Karp | Y |
| 246 | HQ718423 | S0915092_KH | Kratie, Cambodia | Cambodia | 2008 | Human | Y | Karp | Y |
| 247 | HQ718428 | S1020210_KH | KampongCham, Cambodia | Cambodia | 2008 | Human | Y | Karp | Y |
| 248 | HQ718447 | U0215166_KH | Kandal, Cambodia | Cambodia | 2010 | Human | Y | Karp | Y |
| 249 | HQ718448 | 01QNg_VN | QuangNgai, Vietnam | Vietnam | 2009 | Human | Y | Karp | Y |
| 250 | HQ718452 | 05QN_VN | QuangNam, Vietnam | Vietnam | 2009 | Human | Y | Karp | Y |
| 251 | HQ718453 | 06QN_VN | Quang Nam, Vietnam | Vietnam | 2009 | Human | Y | Karp | Y |
| 252 | HQ718455 | 11QY87_VN | Khan Hoa, Vietnam | Vietnam | 2009 | Human | Y | Karp | Y |
| 253 | HQ718456 | 13QY87_VN | Khan Hoa, Vietnam | Vietnam | 2009 | Human | Y | Karp | Y |
| 254 | HQ718458 | 33BVKH_VN | Khan Hoa, Vietnam | Vietnam | 2010 | Human | Y | Karp | Y |
| 255 | KJ001159 | Jin / 2012 | Zhejiang, China | China | 2012 | Human | Y | Karp | Y |
| 256 | AF302983 | HSB1 | Saitama, Japan | Japan | 1997 | Rodent | Y | Karp | Y |
| 257 | AY222634 | TW441 | Kinmen, Taiwan, China | China | 1999 | Rodent | Y | Karp | Y |
| 258 | AY222638 | TW141 | Penghu, Taiwan, China | China | 1986 | Rodent | Y | Karp | Y |
| 259 | AY222639 | TW121 | Lan-Yu, Taiwan, China | China | 1990 | Rodent | Y | Karp | Y |
| 260 | KC693732 | SH245 | Kochi, Japan | Japan | 2007 | Rodent | Y | Karp | Y |
| 261 | AF173048 | Nishino | Gifu, Japan | Japan | 1988 | Human | Y | Boryong | Y |
| 262 | GQ332750 | TPC0701a | Taipei, Taiwan, China | China | 2007 | Human | Y | Boryong | Y |
| 263 | HQ660211 | Inha-B201883-4 | South Korea | South Korea | 2009 | Human | Y | Boryong | Y |
| 264 | JQ898356 | CBNU-9 | South Korea | South Korea | 2009 | Human | Y | Boryong | Y |
| 265 | M63380 | Kuroki | Miyazaki, Japan | Japan | 1981 | Human | Y | Boryong | Y |
| 266 | AY222631 | TW461 | Chengkung, Taiwan, China | China | 1986 | Rodent | Y | Gilliam | Y |
| 267 | AY335819 | Taitung-2 | Taiwan, China | China | 2002 | Human | Y | Gilliam | Y |
| 268 | AY525145 | Hualien-2 | Hualien, Taiwan, China | China | 2003 | Human | Y | Gilliam | Y |
| 269 | AY834393 | Hualien-7 | Hualien, Taiwan, China | China | 2002 | Human | Y | Gilliam | Y |
| 270 | AY856071 | Hualien-9 | Hualien, Taiwan, China | China | 2003 | Human | Y | Gilliam | Y |
| 271 | DQ323174 | Hualien-8 | Hualien, Taiwan, China | China | 2002 | Human | Y | Gilliam | Y |
| 272 | EF213096 | UT125 | Muang, UdornThani, Thailand | Thailand | 2003 | Human | Y | Gilliam | Y |
| 273 | EF213085 | FPW2016 | PhoPra, Tak, Thailand | Thailand | 2004 | Human | Y | Gilliam | Y |
| 274 | GQ332758 | TPC0707a | Taipei, Taiwan, China | China | 2007 | Human | Y | Gilliam | Y |
| 275 | GU120165 | TT02-1 | Taitung, Taiwan, China | China | 2008 | Chigger | Y | Gilliam | Y |
| 276 | HQ718426 | S1006257_KH | KampongCham, Cambodia | Cambodia | 2008 | Human | Y | Gilliam | Y |
| 277 | HQ718429 | S1213056_KH | PhnomPenh, Cambodia | Cambodia | 2008 | Human | Y | Gilliam | Y |
| 278 | HQ718440 | T1015340_KH | Kandal, Cambodia | Cambodia | 2009 | Human | Y | Gilliam | Y |
| 279 | HQ718441 | T1019165_KH | Kratie, Cambodia | Cambodia | 2009 | Human | Y | Gilliam | Y |
| 280 | HQ718444 | T1116116_KH | KohKong, Cambodia | Cambodia | 2009 | Human | Y | Gilliam | Y |
| 281 | HQ718460 | 61QN_VN | Quang Nam, Vietnam | Vietnam | 2009 | Human | Y | Gilliam | Y |
| 282 | KJ001161 | Liu / 2011 | Zhejiang, China | China | 2011 | Human | Y | Gilliam | Y |
| 283 | GU446598 | KM16-2 | Kinmen, Taiwan, China | China | 2009 | Chigger | Y | Gilliam | Y |
| 284 | AY243357 | Hualien-1 | Hualien, Taiwan, China | China | 2002 | Human | Y | Gilliam | Y |
| 285 | GQ332754 | NT0711a | Nantou, Taiwan, China | China | 2007 | Human | Y | Gilliam | Y |
| 286 | GQ332755 | TT0711a | Taitung, Taiwan, China | China | 2007 | Human | Y | Gilliam | Y |
| 287 | AF050669 | Sxh951 | Shanxi, China | China | 1998 | Human | Y | Gilliam | Y |
| 288 | AF173033 | Ikeda | Niigata, Japan | Japan | 1979 | Human | Y | Gilliam | Y |
| 289 | U19903 | Yonchon | South Korea | South Korea | 1989 | Human | Y | Gilliam | Y |
| 290 | DQ485289 | Taiwan CDC Gilliam | Myanmar | Myanmar | 2002 | Human | Y | Gilliam | Y |
| 291 | GU120147 | KM02 | Kinmen, Taiwan | China | 2002 | Chigger | Y | Gilliam | Y |
| 292 | HQ718421 | S0617100_KH | Takeo, Cambodia | Cambodia | 2009 | Human | Y | Gilliam | Y |
| 293 | GQ332753 | TT0705a | Taitung, Taiwan, China | China | 2007 | Human | Y | Gilliam | Y |
| 294 | GU446595 | KM15-1 | Kinmen, Taiwan, China | China | 2009 | Chigger | Y | Gilliam | Y |
| 295 | AF173038 | Taguchi | Gifu, Japan | Japan | 1984 | Human | Y | Kawasaki | Y |
| 296 | JQ898349 | CBNU-2 | South Korea | South Korea | 2009 | Human | Y | Kawasaki | Y |
| 297 | M63383 | Kawasaki | Miyazaki, Japan | Japan | 1981 | Human | Y | Kawasaki | Y |
| 298 | JQ898358 | CBNU-11 | South Korea | South Korea | 2009 | Human | Y | Kawasaki | Y |
| 299 | GQ332756 | PT0712b | Pingtung, Taiwan, China | China | 2007 | Human | Y | Kawasaki | Y |
| 300 | JQ898348 | CBNU-1 | South Korea | South Korea | 2009 | Human | Y | Kawasaki | Y |
| 301 | JQ898360 | CBNU-13 | South Korea | South Korea | 2009 | Human | Y | Kawasaki | Y |
| 302 | JQ898361 | CBNU-14 | South Korea | South Korea | 2009 | Human | Y | Kawasaki | Y |
| 303 | JQ898374 | CBNU-28 | South Korea | South Korea | 2010 | Human | Y | Kawasaki | Y |
| 304 | JQ898376 | CBNU-30 | South Korea | South Korea | 2010 | Human | Y | Kawasaki | Y |
| 305 | AY787232 | Taitung-4 | Taiwan, China | China | 2004 | Human | Y | TA763 | Y |
| 306 | DQ323175 | Hualien-11 | Hualien, Taiwan, China | China | 2004 | Human | Y | TA763 | Y |
| 307 | EF213095 | UT302 | Muang, UdornThani, Thailand | Thailand | 2004 | Human | Y | TA763 | Y |
| 308 | GU120149 | KM04 | Kinmen, Taiwan, China | China | 2008 | Chigger | Y | TA763 | Y |
| 309 | GU120153 | KM08 | Kinmen, Taiwan, China | China | 2008 | Chigger | Y | TA763 | Y |
| 310 | GU120160 | PH03 | Penghu, Taiwan, China | China | 2008 | Chigger | Y | TA763 | Y |
| 311 | GU446613 | OI05-1 | Lan-Yu, Taiwan, China | China | 2009 | Chigger | Y | TA763 | Y |
| 312 | HQ718459 | 45QN_VN | Quang Nam, Vietnam | Vietnam | 2009 | Human | Y | TA763 | Y |
| 313 | AY222630 | TW521 | Lan-Yu, Taiwan, China | China | 1990 | Rodent | Y | TA763 | Y |
| 314 | AY222635 | TW381 | Lan-Yu, Taiwan, China | China | 1990 | Rodent | Y | TA763 | Y |
| 315 | DQ852664 | Hualien-14 | Hualien, Taiwan, China | China | 2004 | Human | Y | TA763 | Y |
| 316 | GQ332752 | NT0707a | Nantou, Taiwan, China | China | 2007 | Human | Y | TA763 | Y |
| 317 | GU120141 | HL02-2 | Hualien, Taiwan, China | China | 2008 | Chigger | Y | TA763 | Y |
| 318 | GU446620 | OI10 | Lan-Yu, Taiwan, China | China | 2009 | Chigger | Y | TA763 | Y |
| 319 | GU446621 | OI011 | Lan-Yu, Taiwan, China | China | 2009 | Chigger | Y | TA763 | Y |
| 320 | HQ718449 | 02QNg_VN | QuangNgai, Vietnam | Vietnam | 2009 | Human | Y | TA763 | Y |
| 321 | U80636 | TA763 | Thailand | Thailand | 1963 | Rodent | Y | TA763 | Y |
| 322 | AY222641 | TWyu11 | Lan-Yu, Taiwan, China | China | 1990 | Rodent | Y | Kato | Classified in Group A with TA763 |
| 323 | AY714315 | Hualien-4 | Hualien, Taiwan, China | China | 2003 | Human | Y | Kato | Classified in Group A with TA763 |
| 324 | EF213087 | FPW1038 | MaeRamat, Tak, Thailand | Thailand | 2004 | Human | Y | Kato | Classified in Group A with TA763 |
| 325 | GU446619 | OI09 | Lan-Yu, Taiwan, China | China | 2009 | Chigger | Y | Kato | Classified in Group A with TA763 |
| 326 | HQ718420 | S0522327_KH | PreyVeng, Cambodia | Cambodia | 2008 | Human | Y | Kato | Classified in Group A with TA763 |
| 327 | U19905 | TA716 | Thailand | Thailand | 1963 | Rodent | Y | Kato | Classified in Group A with TA763 |
| 328 | AF173050 | LF-1 | Malaysia | Malaysia | 1993 | Chigger | Y | Kato | Y |
| 329 | AY636101 | Hualien-3 | Hualien, Taiwan, China | China | 2003 | Human | Y | Kato | Y |
| 330 | AY714316 | Hualien-5 | Hualien, Taiwan, China | China | 2003 | Human | Y | Kato | Y |
| 331 | AY714317 | Hualien-6 | Hualien, Taiwan, China | China | 2002 | Human | Y | Kato | Y |
| 332 | GQ332761 | HC0605a | Hsinchu, Taiwan, China | China | 2006 | Human | Y | Kato | Y |
| 333 | GQ332762 | KM0607b | Kinmen, Taiwan, China | China | 2006 | Human | Y | Kato | Y |
| 334 | GU120143 | HL03-2 | Hualien, Taiwan, China | China | 2008 | Chigger | Y | Kato | Y |
| 335 | GU120148 | KM03 | Kinmen, Taiwan, China | China | 2003 | Chigger | Y | Kato | Y |
| 336 | GU120169 | TT03-2 | Taitung, Taiwan, China | China | 2008 | Chigger | Y | Kato | Y |
| 337 | GU120170 | TT04 | Taitung, Taiwan, China | China | 2008 | Chigger | Y | Kato | Y |
| 338 | GU446608 | OI01-2 | Lan-Yu, Taiwan, China | China | 2009 | Chigger | Y | Kato | Y |
| 339 | GU446611 | OI03-2 | Lan-Yu, Taiwan, China | China | 2009 | Chigger | Y | Kato | Y |
| 340 | GU446612 | OI04 | Lan-Yu, Taiwan, China | China | 2009 | Chigger | Y | Kato | Y |
| 341 | U19904 | TA678 | Thailand | Thailand | 1963 | Rodent | Y | Kato | Y |
| 342 | GU446615 | OI06-1 | Lan-Yu, Taiwan, China | China | 2009 | Chigger | Y | Kato | Y |
| 343 | HQ718424 | S0923259_KH | Kandal, Cambodia | Cambodia | 2008 | Human | Y | Kato | Y |
| 344 | M63382 | Kato | Niigata, Japan | Japan | 1955 | Human | Y | Kato | Y |
| 345 | M63381 | Shimokoshi | Niigata, Japan | Japan | 1980 | Human | Y | Shimokoshi | Y |
| 346 | U80635 | TA686 | Thailand | Thailand | 1963 | Rodent | Y | TA686 | Y |
| 347 | AF173042 | LX-1 | Niigata, Japan | Japan | 1986 | Chigger | Y | Shimokoshi | Classified in Group L away from Shimokoshi_Japan_1980 |
| 348 | AF201834 | Fuji | Obara, Aichi, Japan | Japan | 1998 | Chigger | Y | Shimokoshi | Classified in Group L away from Shimokoshi_Japan_1980 |

**Supplementary table 3**. Information of 24 selected sequences with entire ORF of *TSA56* gene.

| **No.** | **NCBI Accession no.** | **Strain name** | **Isolated location** | **Isolated country** | **Isolated year** | **Isolation host** | **Have entire ORF sequence(Y or N)** | **Genotype** | **Re-grouping result** |
| --- | --- | --- | --- | --- | --- | --- | --- | --- | --- |
| 1 | U80635 | TA686 | Thailand | Thailand | 1963 | Rodent | Y | TA686 | Group A |
| 2 | AY787232 | Taitung-4 | Taiwan, China | China | 2004 | Human | Y | TA763 | Group B |
| 3 | MT258807 | CH01170 | Guangzhou, Guangdong, China | China | 2016 | Human | Y | TA763 | Group B |
| 4 | AY222641 | TWyu11 | Lan-Yu, Taiwan, China | China | 1990 | Rodent | Y | Kato | Group B (Group B-1) |
| 5 | GU446615 | OI06-1 | Lan-Yu, Taiwan, China | China | 2009 | Chiggers | Y | Kato | Group C |
| 6 | HQ718424 | S0923259-KH | Kandal, Cambodia | Cambodia | 2008 | Human | Y | Kato | Group C |
| 7 | GU120169 | TT03-2 | Taitung, Taiwan, China | China | 2008 | Chiggers | Y | Kato | Group D |
| 8 | GU120170 | TT04 | Taitung, Taiwan, China | China | 2008 | Chiggers | Y | Kato | Group D |
| 9 | AY714316 | Hualien-5 | Hualien, Taiwan, China | China | 2003 | Human | Y | Kato | Group E |
| 10 | M63382 | Kato | Niigata, Japan | Japan | 1955 | Human | Y | Kato | Group E |
| 11 | MT258799 | HD01017 | Guangzhou, Guangdong, China | China | 2015 | Human | Y | Kato | Group E |
| 12 | AY243357 | Hualien-1 | Hualien, Taiwan, China | China | 2002 | Human | Y | Gilliam | Group F |
| 13 | GQ332753 | TT0705a | Taitung, Taiwan, China | China | 2007 | Human | Y | Gilliam | Group F |
| 14 | MT258819 | CH01117 | Guangzhou, Guangdong, China | China | 2016 | Human | Y | Gilliam | Group F |
| 15 | M63383 | Kawasaki | Miyazaki, Japan | Japan | 1981 | Human | Y | Kawasaki | Group G |
| 16 | JQ898348 | CBNU-1 | South Korea | South Korea | 2009 | Human | Y | Kawasaki | Group G |
| 17 | M63381 | Shimokoshi | Niigata, Japan | Japan | 1980 | Human | Y | Shimokoshi | Group I |
| 18 | HQ718452 | 05QN-VN | QuangNam, Vietnam | Vietnam | 2009 | Human | Y | Karp | Group J |
| 19 | AY222638 | TW141 | Penghu, Taiwan, China | China | 1986 | Rodent | Y | Karp | Group J |
| 20 | MT258812 | CH01141 | Guangzhou, Guangdong, China | China | 2016 | Human | Y | Karp | Group J |
| 21 | HQ660211 | Inha-B201883-4 | South Korea | South Korea | 2009 | Human | Y | Boryong | Group K |
| 22 | AF173048 | Nishino | Gifu, Japan | Japan | 1988 | Human | Y | Boryong | Group K |
| 23 | AF173042 | LX-1 | Niigata, Japan | Japan | 1986 | Chigger | Y | Shimokoshi | Group L |
| 24 | AF201834 | Fuji | Obara, Aichi, Japan | Japan | 1998 | Chigger | Y | Shimokoshi | Group L |
